# In Search for Multi-Target Ligands as Potential Agents for Diabetes Mellitus and Its Complications—A Structure-Activity Relationship Study on Inhibitors of Aldose Reductase and Protein Tyrosine Phosphatase 1B

**DOI:** 10.3390/molecules26020330

**Published:** 2021-01-10

**Authors:** Rosaria Ottanà, Paolo Paoli, Mario Cappiello, Trung Ngoc Nguyen, Ilenia Adornato, Antonella Del Corso, Massimo Genovese, Ilaria Nesi, Roberta Moschini, Alexandra Naß, Gerhard Wolber, Rosanna Maccari

**Affiliations:** 1 Department of Chemical, Biological, Pharmaceutical and Environmental Sciences, University of Messina, Viale Palatucci, Polo Universitario Annunziata, 98168 Messina, Italy; rottana@unime.it (R.O.); ilenia_adornato@hotmail.it (I.A.); 2 Department of Scienze Biomediche Sperimentali e Cliniche, Sezione di Scienze Biochimiche, University of Firenze, Viale Morgagni 50, 50134 Firenze, Italy; paolo.paoli@unifi.it (P.P.); massimo.genovese@student.unisi.it (M.G.); i.nesi2@student.unisi.it (I.N.); 3 Department of Biology, Biochemistry Unit, University of Pisa, Via S. Zeno, 51, 56123 Pisa, Italy; mario.cappiello@unipi.it (M.C.); antonella.delcorso@unipi.it (A.D.C.); roberta.moschini@unipi.it (R.M.); 4 Molecular Design Lab, Institute of Pharmacy, Freie Universität Berlin, Königin-Luisestr. 2 + 4, 14195 Berlin, Germany; gockyy@zedat.fu-berlin.de (T.N.N.); alexandra.nass@stenon.io (A.N.); gerhard.wolber@fu-berlin.de (G.W.)

**Keywords:** multi-target ligands, diabetes mellitus, aldose reductase, protein tyrosine phosphatase 1B, 4-thiazolidinones, molecular docking

## Abstract

Diabetes mellitus (DM) is a complex disease which currently affects more than 460 million people and is one of the leading cause of death worldwide. Its development implies numerous metabolic dysfunctions and the onset of hyperglycaemia-induced chronic complications. Multiple ligands can be rationally designed for the treatment of multifactorial diseases, such as DM, with the precise aim of simultaneously controlling multiple pathogenic mechanisms related to the disease and providing a more effective and safer therapeutic treatment compared to combinations of selective drugs. Starting from our previous findings that highlighted the possibility to target both aldose reductase (AR) and protein tyrosine phosphatase 1B (PTP1B), two enzymes strictly implicated in the development of DM and its complications, we synthesised 3-(5-arylidene-4-oxothiazolidin-3-yl)propanoic acids and analogous 2-butenoic acid derivatives, with the aim of balancing the effectiveness of dual AR/PTP1B inhibitors which we had identified as designed multiple ligands (DMLs). Out of the tested compounds, **4f** exhibited well-balanced AR/PTP1B inhibitory effects at low micromolar concentrations, along with interesting insulin-sensitizing activity in murine C2C12 cell cultures. The SARs here highlighted along with their rationalization by in silico docking experiments into both target enzymes provide further insights into this class of inhibitors for their development as potential DML antidiabetic candidates.

## 1. Introduction 

In the last decade, the design and discovery of multi-target-directed ligands has emerged as a modern promising strategy to develop new drugs aimed to the treatment of multifactorial diseases, such as diabetes, cancer, infectious diseases, cardiovascular and neurodegenerative disorders. Therapies based on a single-target-directed agent may be insufficient for the management of these complex pathologies, thus combinations of different drugs generally represent therapeutic regimens necessary in order to achieve a higher efficacy. However, combination therapies may involve pharmacokinetics, toxicity or patient compliance problems.

Therefore, recently multi-targeted ligands have attracted great interest in medicinal chemistry as a valuable alternative to combinations of single-targeted drugs. In particular, designed multiple ligands (DMLs) are compounds rationally designed to modulate two or more selected targets simultaneously and, thus, to achieve pharmacological effects favourable for the efficient management of multifactorial pathologies. Growing evidence indicates that DMLs may be endowed with enhanced efficacy and improved safety compared to drug combinations, especially in long-term therapies [1,2,3,4].

A drug design strategy frequently used to obtain DMLs starts from the knowledge of the distinct pharmacophores of agents active on each of the individual targets and consists in the integration of these pharmacophores into a single structure to obtain hybrid molecules. Hybridization should be carried out in such a way that the pharmacophoric elements inserted in a resulting DML maintain the ability to interact with specific sites of the different selected targets, thus producing simultaneously the desired effects. In order to obtain molecules endowed with appropriate drug-like properties, it is often preferred to partially overlap the different pharmacophores, by using or merging them. However, by applying the different possible approaches for the design of DMLs, it is quite likely to achieve lead compounds endowed with high potency towards only one of the desired targets, with less effect on the others; therefore, it is often necessary to carry out a balancing process, using structural modifications of the lead compound, aimed to modulate and balance the activity towards the selected targets until to reach an optimal ratio [1,4].

Recently we have reported an initial investigation on 4-thiazolidinone derivatives active as dual inhibitors of aldose reductase and protein tyrosine phosphatase 1B, in the context of our search for new candidates for the treatment of type 2 diabetes mellitus and its complications [5]. Diabetes mellitus (DM) is a complex chronic metabolic disease which represents a global severe health problem. It has been estimated that currently more than 460 million people are affected by DM worldwide and the prevalence of this disease is expected to increase to about 700 million by 2045 [6,7]. Moreover, it is estimated that about 10% of the total deaths in the world are linked to DM. Glycaemic control is the main goal in DM care and oral antihyperglycaemic drugs or insulin therapy are commonly used for this purpose. However, when not adequately treated, diabetic patients may be chronically exposed to unnatural glucose fluctuations, which induce cell damage and trigger a low-grade systemic inflammation, ultimately causing the onset of severe long-term complications, such as micro/macroangiopathies, neuropathy, nephropathy and cardiovascular dysfunctions. Therefore, to obtain an effective glycaemic control and prevent chronic complications, the standard therapeutic treatment of DM is often represented by drug cocktails. Consequently, it is conceivable that different key pathogenic events involved in DM could be advantageously addressed by DMLs [8,9].

On these bases, in the context of a search for new DMLs as potential antidiabetic agents, we selected as targets aldose reductase (AR) and protein tyrosine phosphatase 1B (PTP1B), two enzymes differently involved in the development of DM and its complications. 

AR (E.C. 1.1.1.21) is an aldo-keto reductase capable to catalyse the reduced nicotinamide adenine dinucleotide phosphate (NADPH)-dependent reduction of a variety of aldehydes, including aldoses. Under hyperglycaemic conditions, such as in DM, an increased flux of glucose through the polyol pathway occurs. AR catalyses the first and rate-limiting step of this metabolic pathway, by reducing glucose to sorbitol which is then oxidized to fructose by sorbitol dehydrogenase. The increased glucose metabolism through the polyol pathway causes osmotic imbalance, decreases antioxidant cellular defence and promotes glycation and inflammatory events, thus leading to tissue damage which is responsible for the development of diabetic chronic complications [10,11]. AR is also capable to reduce reactive unsaturated aldehydes, derived from oxidative stress-induced lipid peroxidation and their glutathione-conjugates, such as 4-hydroxy-2,3-nonenal (HNE), thus participating in the detoxification processes of toxic aldehydes. However, the AR-catalysed reduction of the adduct glutathione-HNE (GS-HNE) generates 3-glutathionyl-1,4-dihydroxynonane (GS-DHN) which is a potent pro-inflammatory molecule; inflammatory events triggered by GS-DHN are strictly related to the development of cellular and vascular damage responsible for diabetic complications. On these bases, AR inhibitors are actively searched to prevent and control the onset and progression of hyperglycaemia-induced pathologies associated to DM and also as potential agents for other inflammatory diseases [11,12,13].

Numerous evidences have undoubtedly demonstrated that PTP1B (E.C. 3.1.3.48) is involved in the development of insulin-resistance, which is a characteristic condition in type 2 DM (T2DM). This protein tyrosine phosphatase acts as a major negative regulator of insulin signalling, by dephosphorylating specific phosphotyrosine residues of the activated insulin receptor and insulin receptor substrate proteins and thus attenuating insulin signalling pathways. PTP1B has been shown to be overexpressed in T2DM and in other pathologies associated with insulin resistance, such as obesity [14,15,16]. The deregulation of this phosphatase is also strictly linked to a chronic low grade inflammation [17], related to insulin resistance and DM. The inhibition or genetic ablation of PTP1B can enhance insulin receptor phosphorylation and improve cellular response to insulin, thus counteracting insulin resistance and increasing glucose uptake into cells [18,19]. Therefore, PTP1B is considered a validated target to develop inhibitors as drug candidates for the treatment of T2DM [20,21,22].

On these bases, both PTP1B and AR can be assumed as attractive molecular targets for the design of DMLs capable to control simultaneously different cellular mechanisms underlying the development of both T2DM and its complications. However, this research is still in its infancy and, so far, only few dual ALR2/PTP1B inhibitors have been reported, among which some natural compounds such as berberine [23,24]. 

**Figure 1:**
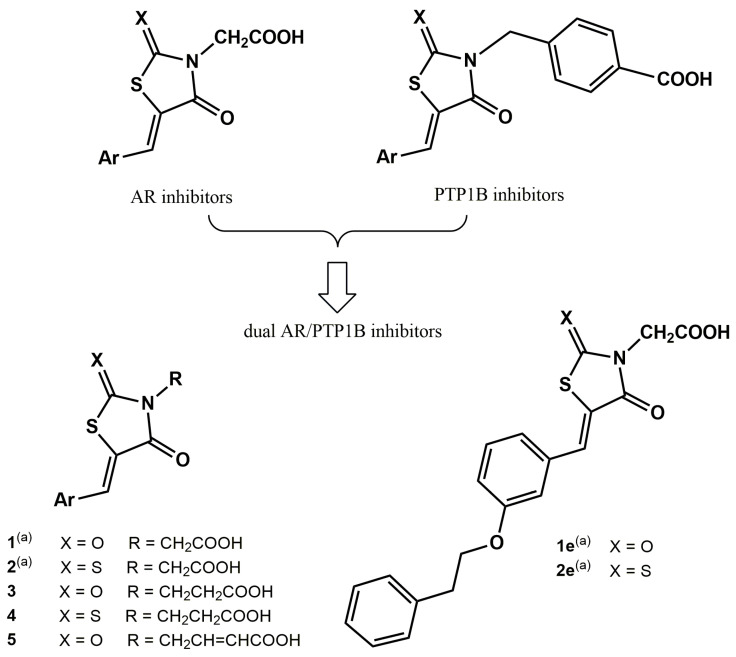
Structures of 4-thiazolidinone derivatives active as inhibitors of aldose reductase (AR) and/or protein tyrosine phosphatase 1B (PTP1B). ^(a)^ Reference [5].

Although these two enzymes belong to different families of proteins, the knowledge of their active sites and the structure-activity relationships (SARs) of inhibitors of each of these targets highlights several shared structural features that enable the design of dual inhibitors by merging the respective pharmacophores. In particular, our previous studies on different 4-thiazolidinone derivatives, which were investigated as AR or PTP1B inhibitors (Figure 1), evidenced that the pharmacophores of both classes of inhibitors comprise a polar head in position 3 of the heterocyclic core, including an acidic or H-bond acceptor group and a lipophilic portion in position 5, containing one or more suitably substituted aromatic moieties [25,26,27,28,29,30,31,32,33,34,35,36,37,38]. 

Therefore, starting from a knowledge-based approach, recently we merged (5-arylidene-4-oxothiazolidin-3-yl)acetic acid derivatives, which are endowed with excellent AR inhibitory activity and 4-[(5-arylidene-4-oxothiazolidin-3-yl)methyl]benzoic acids, which we identified as potent PTP1B inhibitors, to obtain a series of new 4-thiazolidinone derivatives (**1**, **2**, Figure 1) that were evaluated as dual inhibitors of both human AR and PTP1B enzymes [5]. Out of them, we identified two analogues, that is, (4-oxo-5-{[3-(2-phenylethoxy)pheny]lmethylidene}-2-thioxothiazolidin-3-yl)acetic acid (**2e**) and the corresponding 2,4-thiazolidinedione counterpart **1e** (Figure 1), which exhibited potent AR inhibitory effects along with appreciable PTP1B inhibitory capability. Kinetic and in silico docking studies clearly indicated that these compounds behave as reversible inhibitors of both human AR and PTP1B, displaying an uncompetitive and non-competitive mechanism of action, respectively [5]. 

The opportunity to design inhibitors capable to bind non-catalytic regions of both AR and PTP1B is a promising tool for the discovery of new DMLs in this context. Therefore, in order to gain further insights into structural requirements for dual AR/PTP1B inhibition, starting from the SARs acquired from compounds **1**–**2**, we designed and synthesised 3-(5-arylidene-4-oxothiazolidin-3-yl)propanoic acids **3a**–**f** and **4a**–**f** and 4-(5-arylidene-2,4-dioxothiazolidin-3-yl)-2-butenoic acids **5a**–**e** (Figure 1). In these newly-synthesised compounds, we modified the carboxylic chain on N-3, with the aim of balancing the inhibitory effects against the two target enzymes. The elongation of this carboxylic chain might be beneficial to ensure further effective interactions with both target enzymes, as suggested by our previous molecular docking investigation. 

Moreover, a previously conducted shape-based analogue search on known active co-crystallized ligands and de novo designed PTP1B inhibitors yielded over 700 commercially available hits, which were docked into PTP1B catalytic site and the most promising ligands were chosen for in vitro evaluation. Out of fourteen selected virtual hits, 3-{[5-(4-benzyloxyphenyl)methylidene]-2,4-dioxothiazolidin-3-yl}propanoic acid showed a moderate capability to inhibit human PTP1B. Therefore, this compound was assumed as a starting point for hit-to-lead optimization.

The insertion of the 2-butenoic chain of compounds **5a**–**e** was also suggested by our previous investigations on both AR and PTP1B inhibitors [26]. In addition, preliminary assays with 4-[(5-arylidene-4-oxothiazolidin-3-yl)methyl]benzoic acids (Figure 1) against bovine lens AR had revealed no inhibition, probably because the bulky benzoic substituent on the N-3 might prevent the interaction with the AR active site. Now, we decided to assess if 2-butenoic acid derivatives are useful to achieve both PTP1B and AR inhibition since the 2-butenoic residue could be assumed as a simpler open mimetic of the benzoic acid residue capable to interact effectively with PTP1B and, at the same time, could bind to AR. 

In the 5-arylidene moiety of compounds **3**–**5**, the structural features, such as two differently linked aromatic rings that had proven to be effective for dual AR/PTP1B inhibition [5] were maintained (Table 1).

Calculated physicochemical and pharmacokinetic parameters of the synthesised compounds **3**–**5** indicated good drug likeness for these molecules and suggested that high oral bioavailability could be expected for all of them (see Supplementary Table S1). 

In addition, although 2-thioxothiazolidinones **4a**–**d** are present in the literature [39,40,41], to our knowledge none of them had ever been evaluated as AR or PTP1B inhibitor so far.

## 2. Results and Discussion

### 2.1. Chemistry

**Scheme 1:**
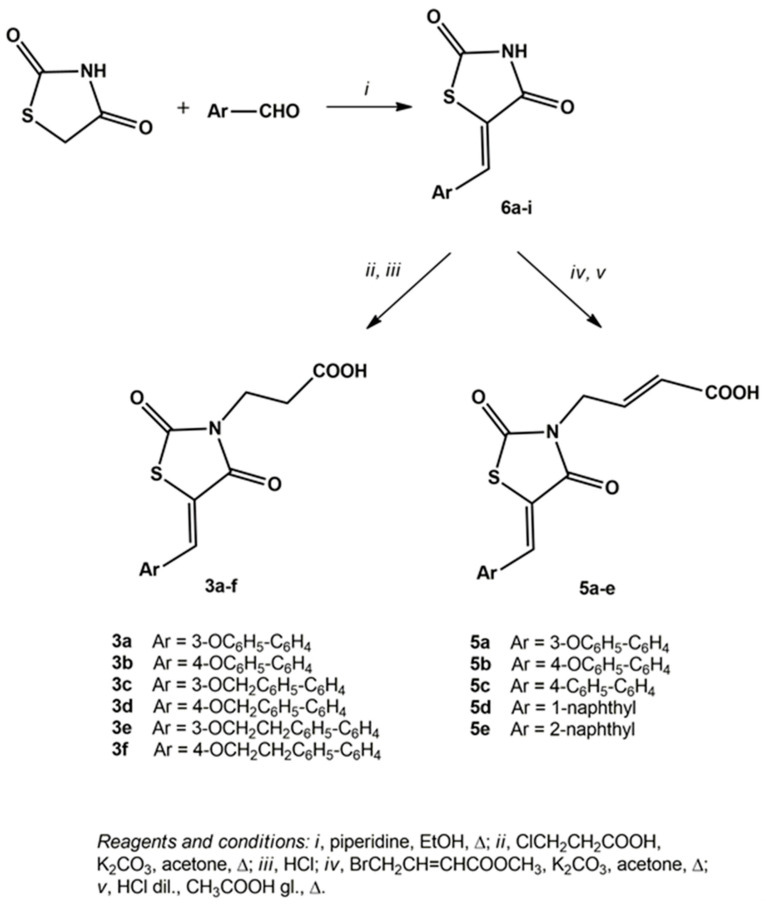
Synthesis of 3-(5-arylidene-2,4-dioxothiazolidin-3-yl)propanoic acids **3a**–**f** and 4-(5-arylidene-2,4-dioxothiazolidin-3-yl)-2-butenoic acids **5a**–**e**.

3-(5-Arylidene-2,4-dioxothiazolidin-3-yl)propanoic acids (**3a**–**f**) and 4-(5-arylidene-2,4-dioxothiazolidin-3-yl)-2-butenoic acids (**5a**–**e**) were prepared through multi-step procedures starting from 5-arylidene-2,4-thiazolidinediones **6a**–**i** (Scheme 1). Precursor compounds **6** were prepared with good yields by the Knoevenagel condensation of commercial 2,4-thiazolidinedione with appropriate aldehydes, in refluxing ethanol and in the presence of piperidine as a base, according to a procedure that we had previously reported [26,37].

Subsequently, the reaction of the appropriate compound **6** with 3-chloropropanoic acid in refluxing acetone and in the presence of potassium carbonate, followed by a work-up in acidic medium and recrystallization from methanol, provided pure 3-(5-arylidene-2,4-dioxothiazolidin-3-yl)propanoic acids (**3a**–**f**) in high yields (Scheme 1). 

The reaction of precursor 2,4-thiazolidinediones **6** with methyl 4-bromocrotonate in refluxing acetone provided 4-(5-arylidene-2,4-dioxothiazolidin-3-yl)-2-butenoic acid methyl esters, which were hydrolysed in an acidic medium to generate the corresponding acids **5a**–**e** (Scheme 1).

**Scheme 2:**
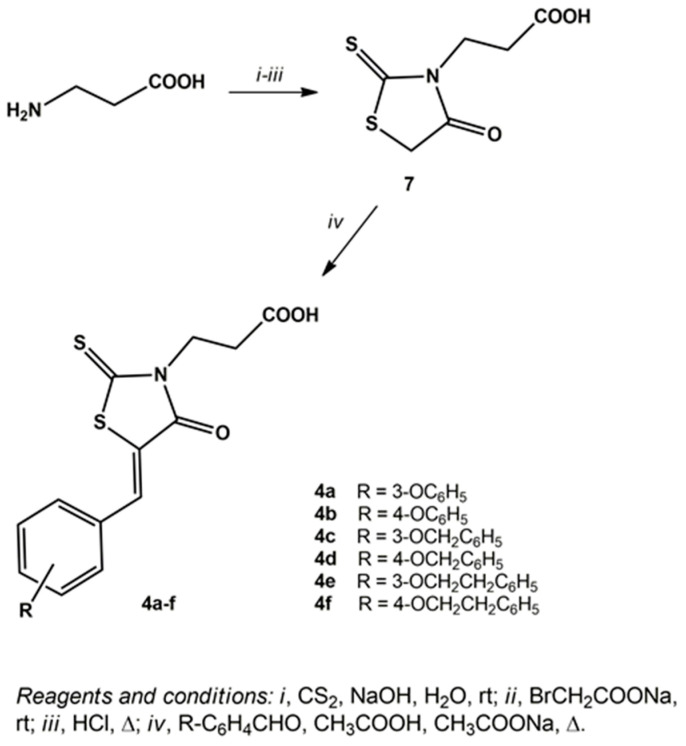
Synthesis of 3-(5-arylidene-4-oxo-2-thioxothiazolidin-3-yl)propanoic acids **4a**–**f**.

The synthetic procedure to obtain 3-(5-arylidene-4-oxo-2-thioxothiazolidin-3-yl)propanoic acids (**4a**–**f**) included the preparation of precursor **7** (Scheme 2). Compound **7** was synthesised by the cyclization of β-alanine with carbon disulphide and sodium bromoacetate, in the presence of sodium hydroxide, in aqueous solution. Subsequently, the desired 3-(5-arylidene-4-oxo-2-thioxothiazolidin-3-yl)propanoic acids (**4a**–**f**) were obtained by the Knoevenagel condensation of 3-(4-oxo-2-thioxothiazolidin-3-yl)propanoic acid (**7**) with the appropriate aldehyde, in refluxing acetic acid in the presence of sodium acetate (Scheme 2).

The structures of compounds **3**–**5** were unambiguously assigned using analytical and ^1^H and ^13^C-NMR spectroscopy data (see Materials and Methods and Supplementary Figures S1–S28). NMR spectroscopy revealed that all synthesised 5-arylidene derivatives **3**–**5** were obtained only as *Z* isomers, analogously to previously investigated 5-arylidene-4-thiazolidinone derivatives which had also been analysed through X-ray crystallography [25,42]. All ^1^H-NMR spectra showed only one singlet attributable to the resonance of the 5-methylidene group, which appeared in the range 7.63–8.57 ppm.

^1^H-NMR spectra of propanoic acids **3** and **4** exhibited two characteristic triplets at 2.57–2.63 ppm and 3.84–4.23 ppm (J = 7.2–7.5 Hz) due to the resonance of the methylene protons of the propanoic chain on N-3. In the spectra of compounds **3e,f** and **4e,f**, two additional triplets (with smaller coupling constant values of 6.8–6.9 Hz) are present at 3.05–3.06 ppm and 4.25–4.29 ppm, originated from the resonance of the ethoxy chain included in the 5-arylidene portion.

In ^1^H-NMR spectra of compounds **5**, the resonance of 2-butenoic chain gave rise to characteristic signals, that is, a doublet at 4.45–4.47 attributable to NCH_2_ protons and two multiplets in the range 5.87–6.83 ppm originated by CH=CH. 

In ^13^C-NMR spectra, the singlets produced by the resonance of the carbonyl groups in the range 165.8–173.1 ppm are diagnostic as well as, in the case of compounds **4**, the signal at 193.7–194.2 ppm attributable to the thiocarbonyl group.

### 2.2. AR and PTP1B Inhibition

The inhibitory effects of compounds **3**–**5** were assessed in vitro against both human recombinant AR, by using L-idose as substrate and epalrestat as the reference drug and human recombinant PTP1B, by using p-nitrophenyl phosphate as substrate and sodium metavanadate as the reference drug. Table 1 reports the data resulting from these assays for the two target enzymes.

Compounds **5a**–**c** had already been evaluated against bovine lens AR, using d,l-glyceraldehyde as a substrate [26]; here, they were assayed against the human enzyme with L-idose as a substrate to obtain comparable data useful for the SAR study. 

All tested 3-(5-arylidene-4-oxothiazolidin-3-yl)propanoic acids **3a**–**f** and **4a**–**f** showed good inhibitory properties against human AR, with IC_50_ values in the mid- and low-micromolar range (Table 1). Their AR inhibitory potency proved to be markedly influenced by the substituents in the positions 2 and 3 of the thiazolidinone scaffold and, to a lesser extent, by the nature of the 5-arylidene moiety. 3-(5-Arylidene-4-oxo-2-thioxothiazolidin-3-yl)propanoic acids **4** exhibited IC_50_ values in the low micromolar range (<10 µM); out of them, **4a** and **4e** proved to be the most potent inhibitors of human AR, with IC_50_ values of 2.2 µM and 2.3 µM, respectively. 2,4-Thiazolidinedione analogues **3** showed the appreciable capability to inhibit the enzyme, especially compounds **3a** and **3c**, which displayed IC_50_ values slightly higher than 10 µM (Table 1). On the whole, 2-thioxo-4-thiazolidinone derivatives **4a**–**f** proved to be from 4- to 12-fold more efficient AR inhibitors than the corresponding 2,4-thiazolidinones **3a**–**f** (Table 1), as already observed for other analogues [32]. 

However, compared with the parent acetic acids (compounds **1, 2** Figure 1), the elongation of the carboxylic chain on N-3 from two to three carbon atoms appeared to be responsible for a significant decrease in AR inhibitory potency for both 3-(5-arylidene-4-oxo-2-thioxothiazolidin-3-yl)propanoic acids **4** and their 2,4-thiazolidinedione analogues **3**. The IC_50_ values obtained for compounds **4a**–**f** revealed that they were from 40-fold to more than 100-fold less potent than acetic acid analogues **2**. Analogously, compounds **3e** and **3f** showed IC_50_ values two orders of magnitude higher than those of the corresponding acetic acid derivatives **1** [5].

The influence exerted on the AR inhibitory potency by the nature of the moiety in position 5 of the heterocyclic core did not appear to be marked; in fact, we observed that the IC_50_ values fall within a relatively narrow range for each series **3** and **4** (Table 1). However, in both series **3** and **4**, compounds bearing a substituent in the *meta* position of the 5-benzylidene ring (**3a**, **3c**, **3e** and **4a**, **4c**, **4e**) exhibited better activity than the *para*-substituted isomers (**3b**, **3d**, **3f** and **4b**, **4d**, **4f**, respectively), confirming a SAR that we had observed in the previously investigated 4-thiazolidinones active as AR inhibitors [25,26,27,28,29,30,31,32]. Among 2-butenoic acid derivatives **5**, compounds **5a** and **5d** exhibited significant capability to inhibit human AR with IC_50_ values of 3.9 μM and 3.7 μM, respectively, whereas the other analogues (**5b**, **5c**, **5e**) produced scarce inhibition at concentrations up to 10 μM (Table 1). It is worthwhile to notice that **5a** had shown very scarce inhibitory activity towards bovine lens AR, in the presence of glyceraldehyde as substrate, whereas **5b** and **5c** were almost inactive towards both bovine and human enzymes with glyceraldehyde and idose as substrate, respectively [26]. These data confirmed previous observations that both the enzymatic source and the substrate used are relevant factors affecting AR inhibitor susceptibility [43,44].

The inhibitory activity towards human PTP1B of compounds **3**–**5** was generally found to be lower than towards human AR. However, all 3-(5-arylidene-4-oxo-2-thioxothiazolidin-3-yl)propanoic acids (**4a**–**f**) and 4-(5-arylidene-2,4-dioxothiazolidin-3-yl)-2-butenoic acids (**5a**–**e**) displayed interesting PTP1B inhibitory properties, with mid-micromolar IC_50_ values (Table 1). Among them, 3-[4-oxo-{5-[4-(2-phenylethoxy)phenyl]methylidene}-2-thioxothiazolidin-3-yl]propanoic acid (**4f**) was shown to be the most effective PTP1B inhibitor (IC_50_ = 12.7 μM). In compounds **4**, we found that selected substituents in the *para* position of the 5-benzylidene ring improved moderately the inhibitory effects, compared to the *meta* isomers.

In addition, it is worthwhile to notice that, in comparison with the previously investigated acetic analogues **2**, the elongation of the carboxylic chain on N-3 generally improved the PTP1B inhibitory activity, as demonstrated by propanoic derivatives **4**; in fact, we found that compounds **4a**, **4b**, **4d** and **4f** were from 2- to 4-fold more potent PTP1B inhibitors than their acetic counterparts [5]. On the other hand, compared with the corresponding 2-thioxo-4-thiazolidinones **4**, the PTP1B inhibitory ability of 2,4-thiazolidinediones **3** was shown to be significantly lower (Table 1), highlighting that 3-(4-oxo-2-thioxothiazolidin-3-yl)]propanoic moiety is more favourable for PTP1B inhibition. However, out of the tested 2,4-thiazolidinedione derivatives, the replacement of the propanoic chain on N-3 (compounds **3**) with a 2-butenoic residue (compounds **5**) provided a gain in potency (**5a**, **5b** versus **3a**, **3b**) and allowed us to identify interesting PTP1B inhibitors. 

Compounds **4a**, **4e** and **4f**, which exhibited appreciable dual AR/PTP1B inhibitory activity, were selected to be further studied for their kinetic behaviour on both target enzymes and on cultured cells. 

### 2.3. Kinetic Studies

Compounds **4a**, **4e** and **4f** behaved as reversible inhibitors toward both AR and PTP1B enzymes. In fact, in the case of AR more than 90% of enzyme activity was recovered after removal of the inhibitor upon extensive dialysis (see Materials and Methods for details). Similar results were obtained with PTP1B (Supplementary Figure S29). 

The evaluation of apparent dissociation constants *K_i_* and *K’_i_* for compounds **4a, 4e** and **4f** (Table 2 and Supplementary Figures S30–S32) indicated **4e** as the most potent inhibitor toward AR. The characterization of the mechanism of action of the three selected compounds revealed for **4a** a mixed-type inhibition, with a slight preference toward the EIS complex (*K’_i_* approximately 4-fold lower than *K_i_*) (Figure S30). On the other hand, both **4e** (Figure S31) and **4f** (Figure S32) resulted to behave essentially as uncompetitive inhibitors (*K_i_* values at least ten-fold higher concerning the corresponding *K’i*).

As far as concerned PTP1B, kinetic analyses revealed that both compounds **4a** and **4f** behave as mixed-type non-competitive inhibitors. Indeed, in both cases, experimental points describing the PTP1B catalytic rate in the presence of increasing concentrations of these compounds were fitted by straight lines intersecting one each other in a point located in the left quadrant (Supplementary Figures S33 and S35). Moreover, we observed that both compounds caused an increase of the Km value (Figures S33 and S35B) and a decrease of Vmax. (Figures S33 and S35C). However, kinetic behaviour of compound **4e** appeared different, as demonstrated by the fact that experimental points described straight lines intersecting one each other’s in a point located on the *Y*-axis (Figure S34A). According to these data, we observed that the Km value increased (Figure S34B), while Vmax did not change with increasing concentration of compound **4e** (Figure S34C); therefore, **4e** behaved as a competitive inhibitor

### 2.4. AR and PTP1B Docking Experiments

Docking studies of selected AR/PTP1B dual inhibitors **4a**, **4e** and **4f** were carried out to evaluate their binding mode with both target enzymes and rationalize the observed inhibition mechanisms. In accordance with the kinetic studies that revealed **4a** as a mixed AR inhibitor, whereas **4e** and **4f** behaved as uncompetitive AR inhibitors, all three compounds showed consistent docking poses with the AR-idose complex, whereas only **4a** revealed plausible docking poses when docked into the catalytic site of AR.

**Figure 2:**
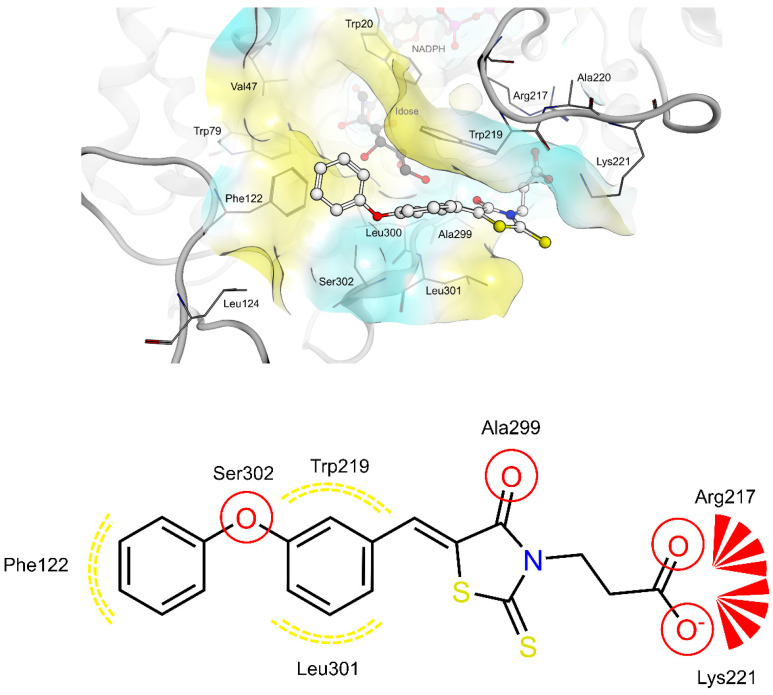
(**Top**): 3D depiction of the selected pose of **4a** bound to the AR-idose complex. Blue surface color: Hydrophilic, Yellow surface color: Lipophilic. (**Bottom**): 2D depiction of protein-ligand interactions of **4a** bound to the AR-idose complex. Red circles: hydrogen bond acceptor, Red stars: Anionic interaction, Yellow dotted lines: Lipophilic contacts.

**Figure 3:**
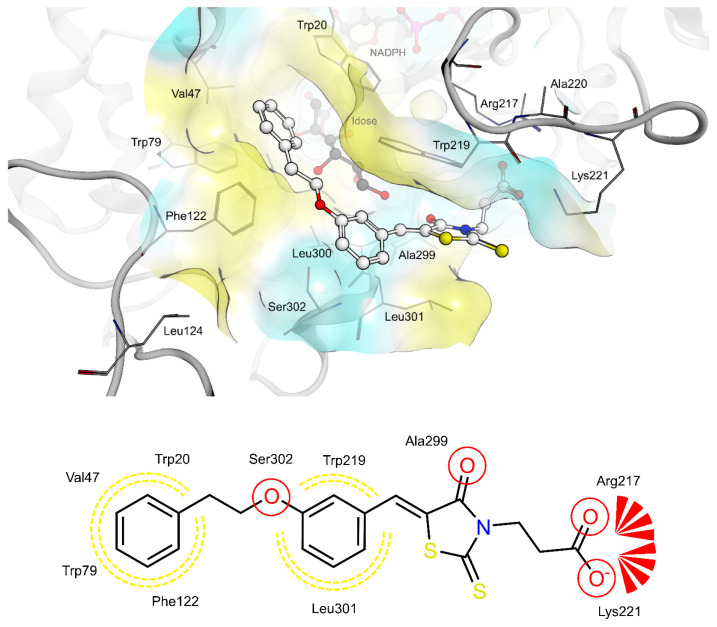
(**Top**): 3D depiction of the selected pose of **4e** bound to the AR-idose complex. Blue surface color: Hydrophilic, Yellow surface color: Lipophilic. (**Bottom**): 2D depiction of protein ligand interactions of **4e** bound to the AR-idose complex. Red circles: hydrogen bond acceptor, Red stars: Anionic interaction, Yellow dotted lines: Lipophilic contacts.

**Figure 4:**
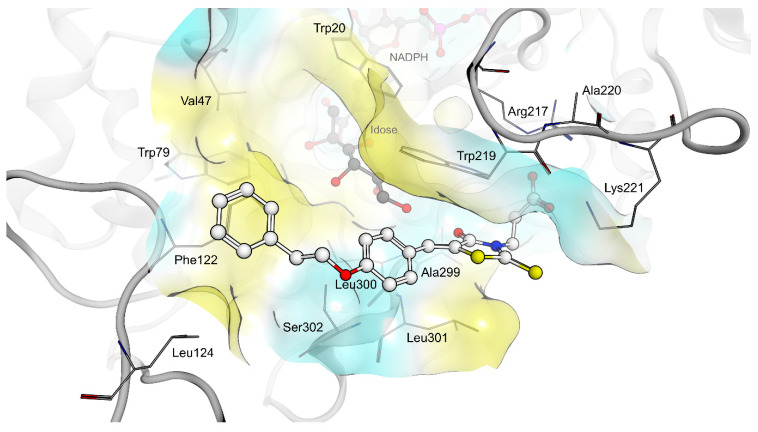

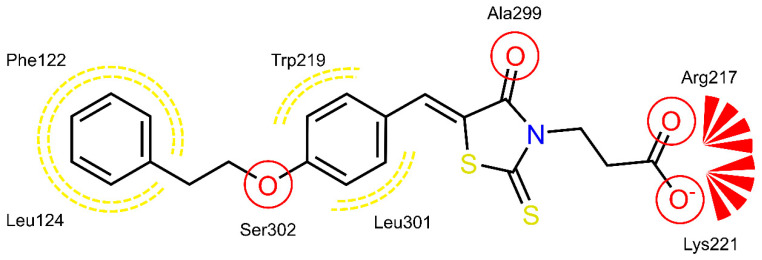
(**Top**): 3D depiction of the selected pose of **4f** bound to the AR-idose complex. Blue surface color: Hydrophilic, Yellow surface color: Lipophilic. (**Bottom**): 2D depiction of protein ligand interactions of **4f** bound to the AR-idose complex. Red circles: hydrogen bond acceptor, Red stars: Anionic interaction, Yellow dotted lines: Lipophilic contacts.

**Figure 5:**
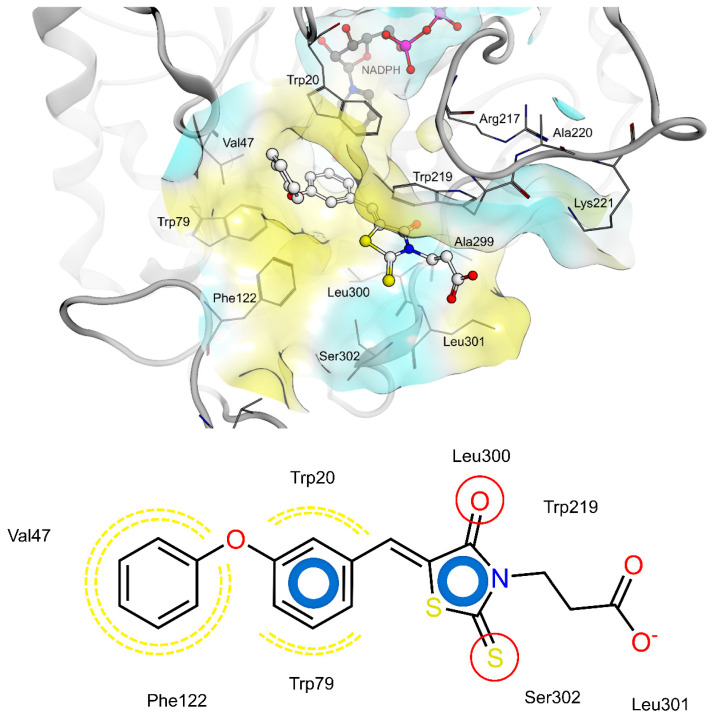
(**Top**): 3D depiction of the selected pose of **4a** bound to the catalytic center AR. Blue surface color: Hydrophilic, Yellow surface color: Lipophilic. (**Bottom**): 2D depiction of protein ligand interactions of **4a** bound to the catalytic center of AR. Red circles: hydrogen bond acceptor, Yellow dotted lines: Lipophilic contacts, Blue donuts: aromatic ring interaction.

The shared propionic acid moiety of **4a**, **4e** and **4f** was located outside of the AR catalytic site, in which L-idose is bound and showed ionic and hydrogen bonding interactions with Arg217 and Lys221 (Figure 2, Figure 3 and Figure 4). Hydrogen bonding interactions can be observed for the carbonyl group in position 4 of the thiazolidinone scaffold with the backbone of Ala299 and the bridging ether oxygen between the two phenyl rings with Ser302. The thiocarbonyl group in position 2 was adjacent to Leu301 and pointed towards the solvent. The 5-benzylidene ring of the compounds formed lipophilic contacts with Trp219 and Leu301. As shown in our previous study [5], a lipophilic moiety in the *meta* position of the 5-benzylidene ring is favourable over a *para* substitution due to the required bent that simultaneously allowed the ligands both to form hydrogen bonding with Arg217 and Lys221 and to fit the lipophilic pocket (Trp20, Val47, Trp79, Phe122) above the idose-bound competitive binding site. The phenylethoxy moiety in the *meta* position of the 5-benzylidene ring (**4e**, Figure 3) established these interactions, whereas the same moiety in the *para* position (**4f**, Figure 4) was more exposed to the solvent and interacted only with Phe122 and Leu124 located outside of the lipophilic pocket. The better fit of **4e** compared to **4f** is in agreement with the AR inhibitory potency trend (IC_50_ = 2.3 μM vs. IC_50_ = 5.3 μM). The replacement of the phenylethoxy group in the meta position of the 5-benzylidene ring with a phenoxy one (compound **4a**) resulted in a detrimental factor for binding to the lipophilic pocket of the AR-idose complex, which is attributable to the shorter linker between the two phenyl groups. However, in the case of **4a** a better fitting of the shorter substituent into the AR narrow catalytic centre came out (Figure 5). This may explain the comparable efficiency of **4a** and **4e**. On the other hand, **4e** and **4f** docked into the catalytic centre revealed unfavorable docking poses where the terminal phenyl group is evidently solvent exposed, thus confirming the observed uncompetitive mode of action. A recent analysis from Balestri et al. indicated rational binding modes to the catalytic centre of AR for different substrates (L-idose, HNE and GSHNE) binding [45]. The aldehyde moiety of all three substrates shows simultaneous hydrogen bonding to His110 and Tyr48. These interactions were established for **4a** neither in our proposed binding mode nor in the other generated docking poses. The reason for this is the rigidity and size of **4a** compared to AR substrates. The diphenylether moiety of **4a** is located slightly above the catalytic centre, embedded between lipophilic residues Trp20, Val47, Trp79 and Phe122 (Figure 5). Interestingly, we observed two aromatic ring interactions, where the thioxothiazolidinone moiety interacted with Trp219 and the 5-benzylidene ring with Trp20. Furthermore, the thiocarbonyl group in position 2 of the thiazolidinone scaffold established hydrogen bonding to Ser302 and the carbonyl group in position 4 to the backbone of Leu300. These observations lead to the conclusion that **4a** shows a different binding mode compared to the AR substrates analysed by Balestri et al. [45] and that direct interaction with Tyr48 and His110 might not be mandatory for competitive binding to the catalytic site of AR.

PTP1B kinetic studies showed that **4a** and **4f** behave as mixed non-competitive inhibitors, whereas **4e** behaves as a pure competitive inhibitor. Therefore, we performed docking studies into both the catalytic binding pocket and the previously investigated allosteric binding pocket of PTP1B [5].

**Figure 6:**
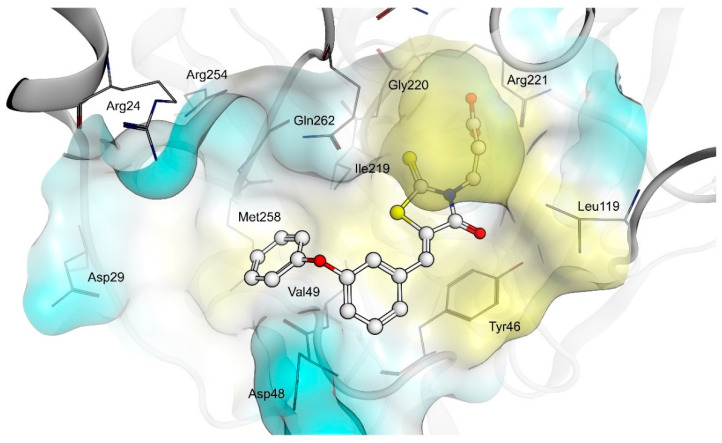

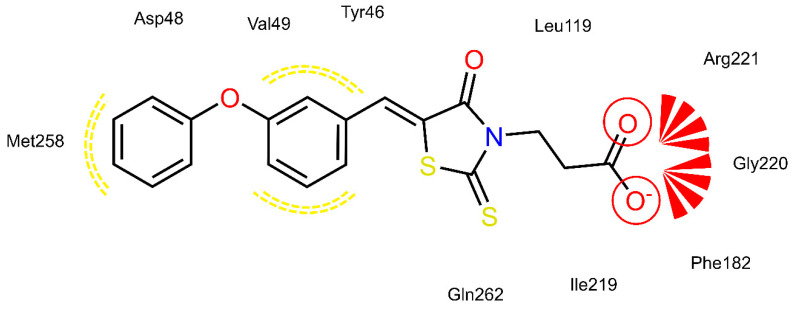
(**Top**): 3D depiction of the selected pose of **4a** bound to the catalytic center of PTP1B. Blue surface color: Hydrophilic, Yellow surface color: Lipophilic. (**Bottom**): 2D depiction of protein-ligand interactions of **4a** bound to the catalytic center of PTP1B. Red circles: hydrogen bond acceptor, Red stars: Anionic interaction, Yellow dotted lines: Lipophilic contacts.

**Figure 7:**
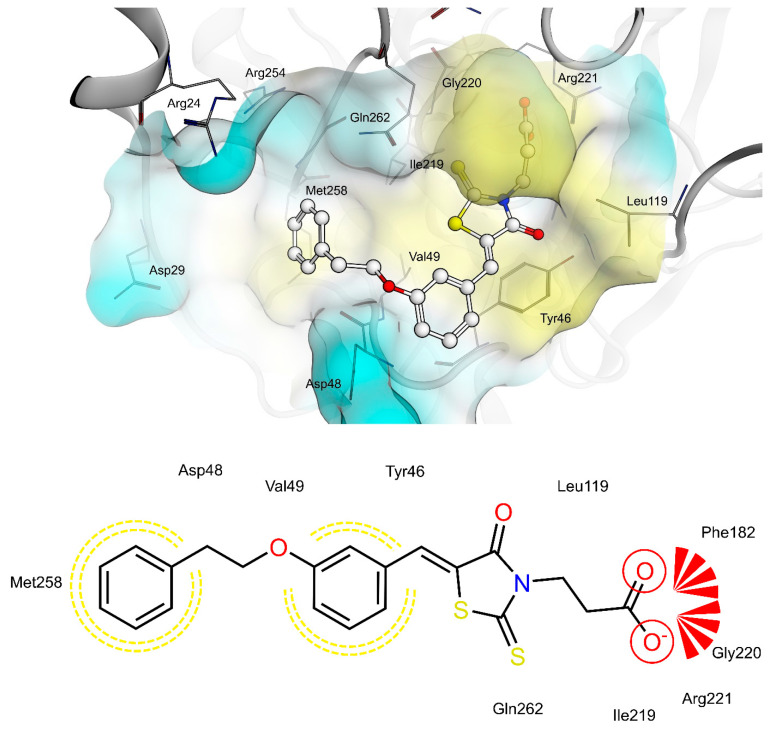
(**Top**): 3D depiction of the selected pose of **4e** bound to the catalytic center of PTP1B. Blue surface color: Hydrophilic, Yellow surface color: Lipophilic. (**Bottom**): 2D depiction of protein-ligand interactions of **4e** bound to the catalytic center of PTP1B. Red circles: hydrogen bond acceptor, Red stars: Anionic interaction, Yellow dotted lines: Lipophilic contacts.

**Figure 8:**
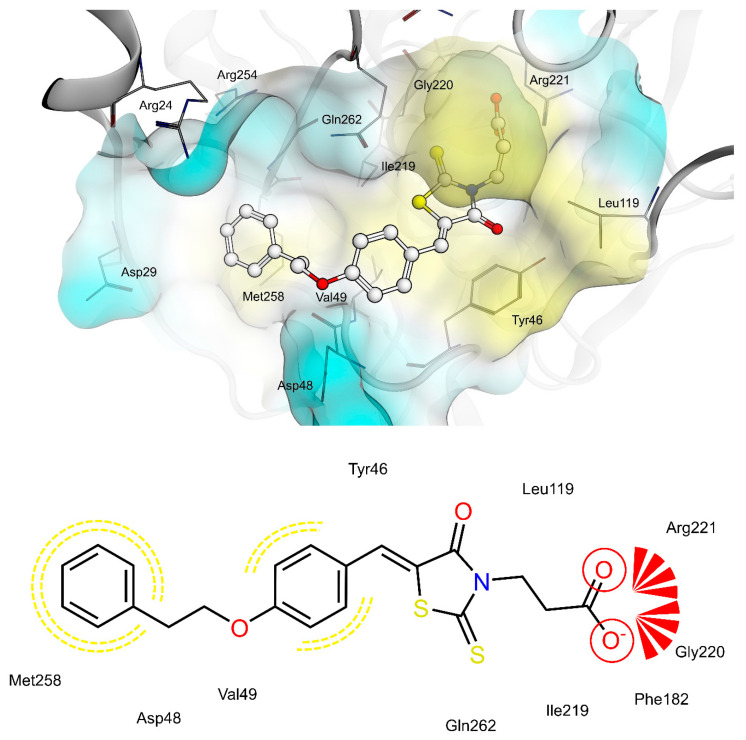
(**Top**): 3D depiction of the selected pose of **4f** bound to the catalytic center of PTP1B. Blue surface color: Hydrophilic, Yellow surface color: Lipophilic. (**Bottom**): 2D depiction of protein-ligand interactions of **4f** bound to the catalytic center of PTP1B. Red circles: hydrogen bond acceptor, Red stars: Anionic interaction, Yellow dotted lines: Lipophilic contacts.

Docking of **4a**, **4e** and **4f** into the catalytic binding site of PTP1B (Figure 6, Figure 7 and Figure 8) revealed hydrogen bonding of the propionic acid moiety to Arg221 and the backbones of Phe182, Ile219 and Gly220. Lipophilic contacts between the 5-benzylidene ring and Tyr46 and Val49 were observed for all three compounds. The terminal phenyl ring of **4a** is embedded into the lipophilic environment consisting of Val49, Ile219 and Met258, whereas the longer 2-ethoxy linker of compounds **4e** and **4f** allowed the terminal phenyl ring to reach out residues Arg24, Arg254 of the adjacent non-catalytic secondary aryl-phosphate binding site [46]. Additionally, in the same binding site, docking poses of **4e** were less consistent compared to **4a** and **4f**, revealing binding poses where the terminal phenyl ring is adjacent to Asp48 and more exposed to the solvent. Therefore, a longer linker on the 5-benzylidene ring (**4e** and **4f**) appeared to be not advantageous for binding at the catalytic region of PTP1B, when in *meta* position, which could explain the lower inhibitory potency of **4e** than **4f**.

**Figure 9:**
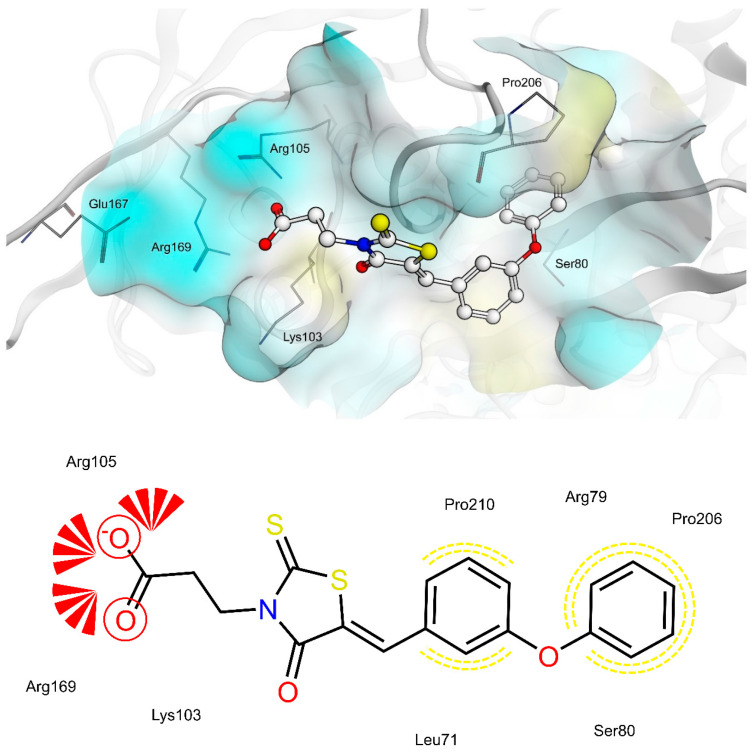
(**Top**): 3D depiction of the selected pose of **4a** bound to the allosteric binding site of PTP1B. Blue surface color: Hydrophilic, Yellow surface color: Lipophilic. (**Bottom**): 2D depiction of protein-ligand interactions of **4a** bound to the allosteric binding site of PTP1B. Red circles: hydrogen bond acceptor, Red stars: Anionic interaction, Yellow dotted lines: Lipophilic contacts.

**Figure 10:**
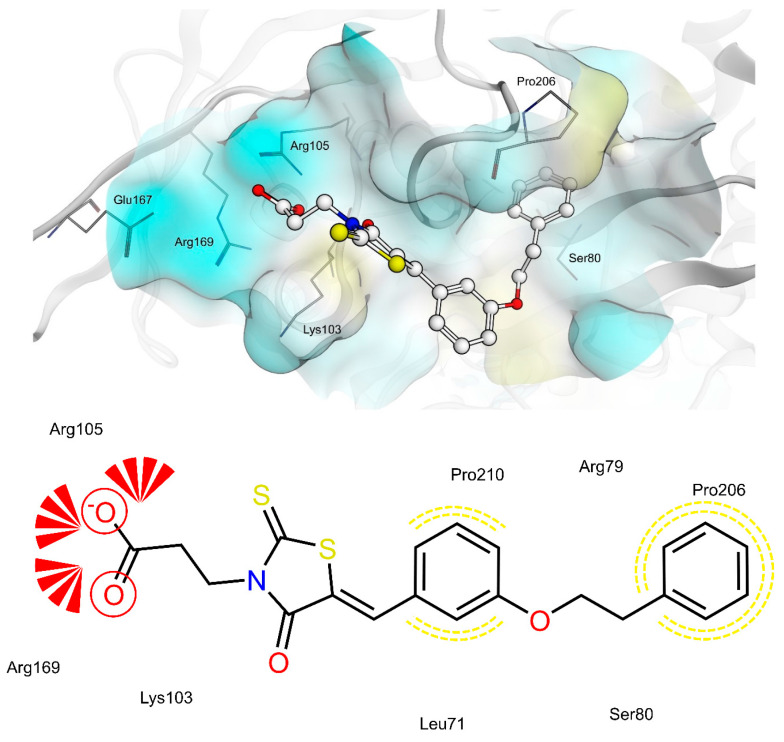
(**Top**): 3D depiction of the selected pose of **4e** bound to the allosteric binding site of PTP1B. Blue surface color: Hydrophilic, Yellow surface color: Lipophilic. (**Bottom**): 2D depiction of protein-ligand interactions of **4e** bound to the allosteric binding site of PTP1B. Red circles: hydrogen bond acceptor, Red stars: Anionic interaction, Yellow dotted lines: Lipophilic contacts.

**Figure 11:**
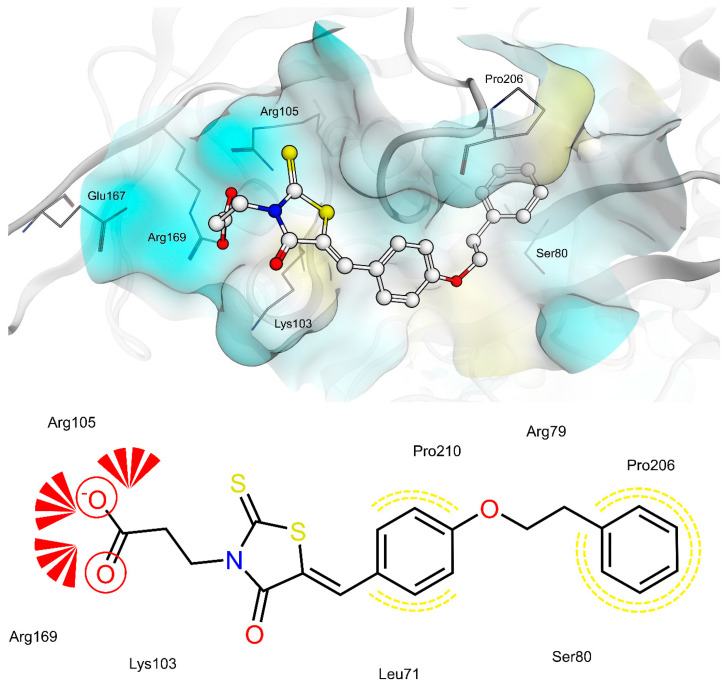
(**Top**): 3D depiction of the selected pose of **4f** bound to the allosteric binding site of PTP1B. Blue surface color: Hydrophilic, Yellow surface color: Lipophilic. (**Bottom**): 2D depiction of protein-ligand interactions of **4f** bound to the allosteric binding site of PTP1B. Red circles: hydrogen bond acceptor, Red stars: Anionic interaction, Yellow dotted lines: Lipophilic contacts.

Docking of **4a**, **4e** and **4f** into the previously described allosteric binding site of PTP1B [5] (Figure 9, Figure 10 and Figure 11) revealed for all three ligands comparable hydrogen bonding interactions of the propionic acid moiety to Lys103, Arg105 and Arg169 and lipophilic contacts of the 5-benzylidene ring to Pro210. The shorter linker between the two phenyl rings of **4a** did not allow the carboxyl group to bind to Lys103, Arg105 and Arg169 by hydrogen bonding and, simultaneously, the terminal phenyl ring to reach the lipophilic pocket consisting of Pro206 and surrounded by Arg79 and Ser80. The longer ethoxy linker of **4f** enabled both hydrogen bonding and lipophilic contacts at the same time. This structural feature could explain the higher inhibitory potency of **4f** (IC_50_ = 12.7 μM) compared to **4a** (IC_50_ = 34.1 μM). Furthermore, the propionic acid in combination with a meta substitution of the 5-benzylidene ring of **4e** made the 5-benzylidene ring more exposed to the solvent, thus resulting in a lower affinity for this allosteric site of **4e** compared to **4a** and **4f**. On the whole, these findings were in agreement with the kinetic study and could further explain the difference in inhibitory potency between **4e** and **4a**, **4f**.

### 2.5. Ex Vivo Assays

**Figure 12:**
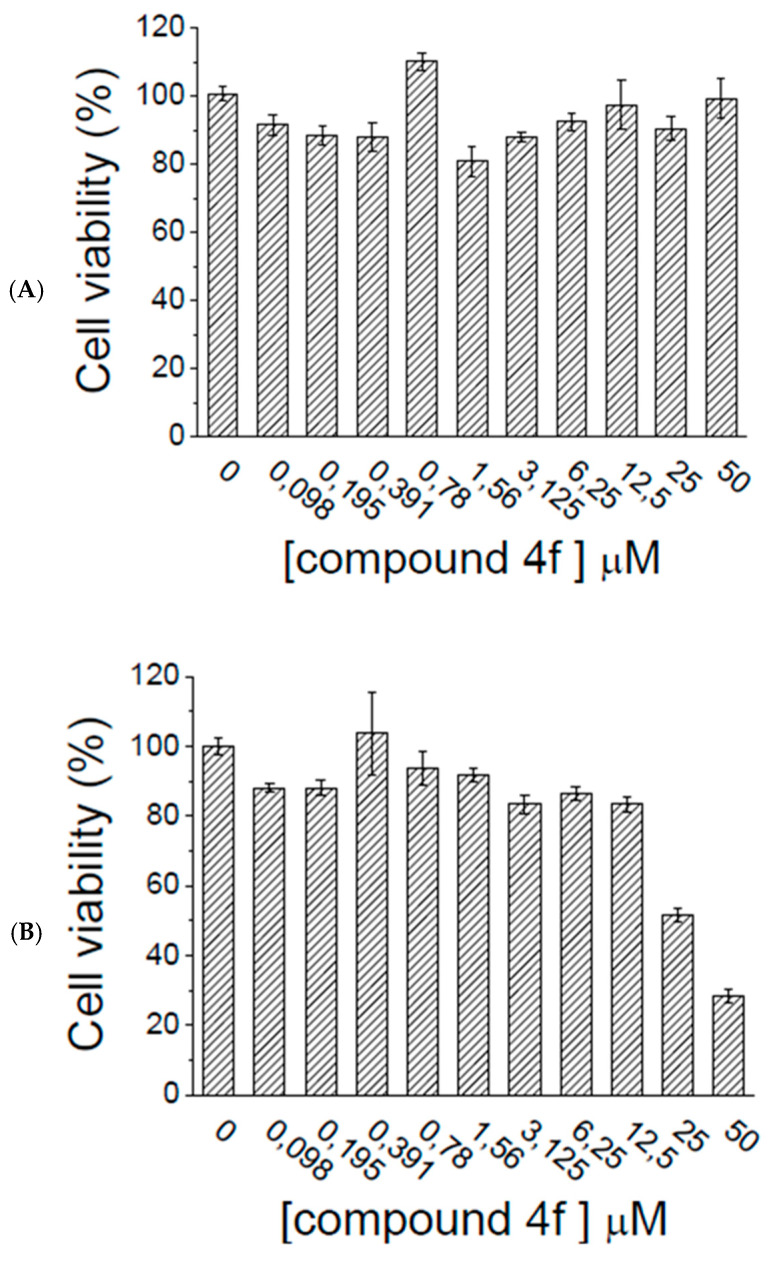
Viability assay. HepG2 cells (**A**) and C2C12 cells (**B**) were incubated in the presence of increasing concentrations (μM) of compounds **4f** for 48 h. After this time, cells viability was assessed using MTT assay. Data obtained were normalized respect to control test. Data showed in the figure represent the mean ± S.E.M. (*n* = 4).

We evaluated the effects of selected compound **4f** on HepG2 and C2C12 cells. Cytotoxicity of the tested compound was determined using MTT assay. Data reported in Figure 12 show that **4f** was well tolerated by both human HepG2 and murine C2C12 cells, as demonstrated by the fact that a reduction of cell viability was observed just when cells (C2C12) were incubated with the highest concentration of compounds (50 μM).

**Figure 13:**
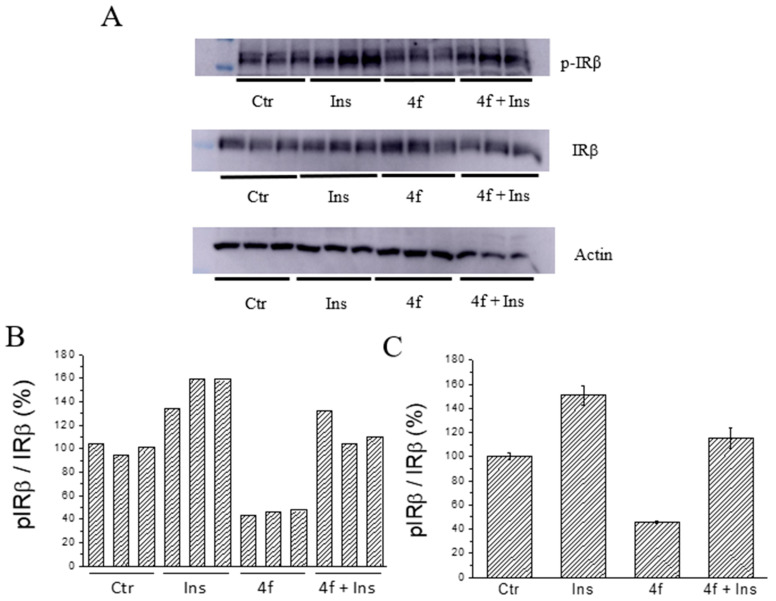
Phosphorylation levels of the insulin receptor in HepG2 cells. Cells were starved for 24 h and then treated for 90 min with compound **4f** (final concentration 25 μM). After this time, cells were washed with PBS and stimulated with insulin (10 nM) for 30 min. Then, cells were washed with cold PBS, lysed and cell extracts analysed by western blot to evaluate insulin receptor phosphorylation levels. The intensity of actin bands was used as a loading control. All tests were carried out in triplicate (*n* = 3). (**A**): western blots. (**B**,**C**): quantification of pIRβ/IRβ ratio.

**Figure 14:**
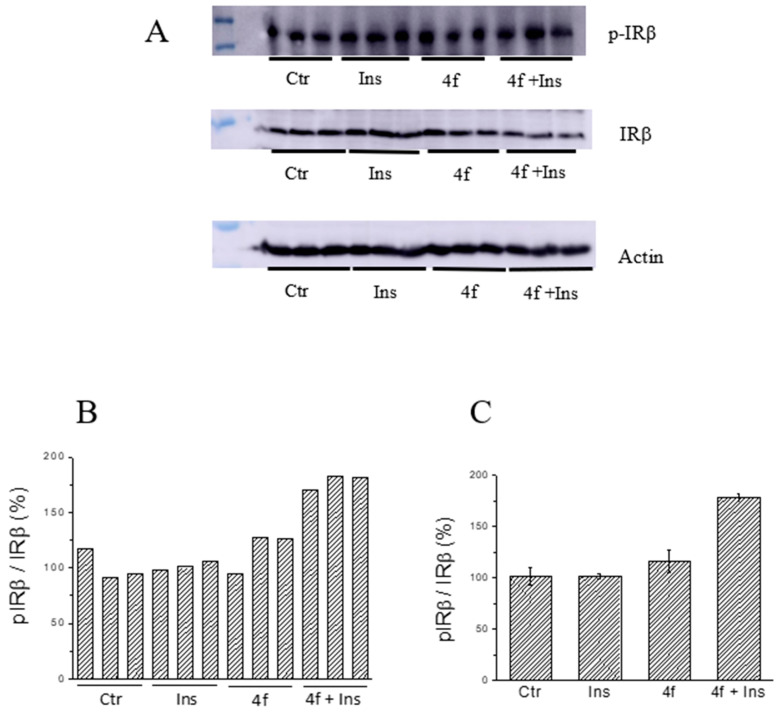
Phosphorylation levels of the insulin receptor in C2C12 cells. Cells were starved for 24 h and then treated for 90 min with compound **4f** (final concentration 25 μM). After this time, cells were washed with PBS and stimulated with insulin (10 nM) for 30 min. Then, cells were washed with cold PBS, lysed and cell extracts analysed by western blot to evaluate insulin receptor phosphorylation levels. The intensity of actin bands was used as a loading control. Each test was carried out in triplicate (*n* = 3). (**A**): western blots. (**B**,**C**): quantification of pIRβ/IRβ ratio.

The insulin mimetic/sensitizing activity of compound 4f was evaluated on both HepG2 and C2C12 cells. Starved HepG2 and C2C12 cells were incubated in the presence of compound **4f** (25 μM) for 90 min and after stimulated or not with insulin (10 nM). Then, cells extracts were analysed to evaluate phosphorylation levels of the insulin receptor. Results of the experiments were showed in Figure 13 and Figure 14.

It is interesting to note that compound **4f** showed a different effect on liver and muscle cells. As far as concerned HepG2 cells, we observed that the treatment with compound **4f** caused a marked reduction of basal phosphorylation levels of IRβ (Figure 13B,C). However, after stimulation with insulin, IRβ phosphorylation levels increase in both control and treated cells, although a relatively higher increase has been observed in the latter. In fact, we calculated that the level of IRβ phosphorylation increases by 39% and 260%, respectively, in the control and in the cells treated with compound **4f**. This finding suggests that the treatment with compound **4f** can make the insulin receptor “more activatable” than that of untreated cells. At the same time, we observed that treating C2C12 cells with compound **4f** did not appreciably affect basal IR phosphorylation levels but significantly improved insulin activity. According to this hypothesis, we found that IRβ phosphorylation levels resulted to be significantly higher in treated cells than in those treated with insulin alone (Figure 14B,C). Taken together, these findings suggest that compound **4f** behaves as an insulin-sensitizing agent in both the liver and muscle cells.

## 3. Materials and Methods

### 3.1. Chemistry

Melting points were recorded on a Kofler hot-stage apparatus and are uncorrected. TLC controls were carried out on precoated silica gel plates (F 254 Merck). Rf values were determined by using appropriate mixtures of diethyl ether/*n*-hexane as eluent. Combustion analyses (C, H, N), determined by means of a C. Erba mod. 1106 elem. Analyzer, were within ±0.4% of the theoretical values. ^1^H and ^13^C-NMR spectra were recorded on a Varian 500 MHz spectrometer operating at 499.74 and 125.73 MHz, respectively. Chemical shifts δ are given in ppm and coupling constants are expressed in Hz. All the spectra were phased, baseline was corrected where necessary and CDCl_3_ or DMSO-*d*_6_ signals were used as a reference for both ^1^H and ^13^C spectra. All exchangeable protons were confirmed by addition of D_2_O. Unless stated otherwise, all materials were obtained from commercial suppliers and used without further purification. 3-(2-Phenylethoxy)benzaldehyde and 4-(2-phenylethoxy)benzaldehyde were synthesised according to the procedure reported in ref. 37. Compounds **3d**, **4a**, **4c**, **4d**, **4f** and **7** are commercially available; however, we synthesised them according to the procedure described below. The purity of synthetic compounds was established as ≥ 95% by combustion analysis.

#### 3.1.1. General Procedure for the Synthesis of 5-arylidene-2,4-dioxothiazolidinones **6a**–**i**

A mixture of 2,4-thiazolidinedione (1.17 g, 10 mmol), the appropriate aldehyde (10 mmol) and piperidine (0.68 g, 8 mmol) in ethanol (70 mL) was refluxed for 24 h. The crude mixture was poured into H_2_O acidified with AcOH (pH 3–4) to give a crude solid which was recrystallized from methanol to provide the corresponding pure 5-arylidene-2,4-thiazolidinedione **6**. Chemical-physical and spectroscopic data of compounds **6a**–**i** were reported in Refs. [26,37].

#### 3.1.2. General Procedure for the Synthesis of 3-(5-arylidene-2,4-dioxothiazolidin-3-yl)propanoic Acids **3a**–**f**

A mixture of 5-arylidene-2,4-thiazolidinedione **6** (1.68 mmol), 3-chloropropanoic acid (0.365 g, 3.36 mmol) and potassium carbonate (0.93 g, 6.73 mmol) in anhydrous acetone (40 mL) was refluxed for 24 h. The solvent was evaporated under reduced pressure. The solid residue was poured into H_2_O, acidified with HCl 6M (pH 3) and stirred until CO_2_ disappearance. The solid was filtered off, washed with H_2_O and recrystallized from methanol to provide pure compounds **3**.

*3-{(5Z)-[2,4-Dioxo-5-(3-phenoxyphenyl)methylidene]thiazolidin-3-yl}propanoic acid* (**3a**). Yield 66% (409.6 mg); m.p. 177–180 °C; ^1^H-NMR (DMSO-*d*_6_): δ 2.59 (t *J* = 7.5 Hz, 2H, CH_2_COOH); 3.84 (t *J* = 7.5 Hz, 2H, NCH_2_); 7.09 (m, 2H, arom); 7.13 (m, 1H, arom); 7.19–7.23 (m, 2H, arom); 7.37 (m, 1H arom); 7.44 (m, 2H, arom), 7.54 (m, 1H, arom); 7.90 (s, 1H, methylidene); 12.45 (s, 1H, COOH). ^13^C-NMR (DMSO-*d*_6_): δ 32.0 (CH_2_), 38.0 (CH_2_), 119.5 (CH), 119.9 (CH), 120.8 (C), 122.8 (C), 124.8 (CH), 125.4 (CH), 130.8 (CH), 131.6 (CH), 132.7 (CH), 135.4 (CH), 156.3 (C), 158.2 (C), 165.8 (C), 167.4 (C), 172.4 (C). Anal. (C_19_H_15_NO_5_S) calcd: C 61.78; H 4.09; N 3.97; found: 61.92; H 4.03; N 3.99.

*3-{(5Z)-[2,4-Dioxo-5-(4-phenoxyphenyl)methylidene]thiazolidin-3-yl}propanoic acid* (**3b**). Yield 60% (372.3 mg); m.p. 198–202 °C; ^1^H-NMR (DMSO-*d*_6_): δ 2.60 (t J = 7.5 Hz, 2H, CH_2_COOH); 3.86 (t *J* = 7.5 Hz, 2H, NCH_2_); 7.11 (m, 4H, arom); 7.24 (m, 1H, arom); 7.45 (m, 2H, arom); 7.64 (m, 2H arom); 7.90 (s, 1H, methylidene); 12.45 (s, 1H, COOH). ^13^C-NMR (DMSO-*d*_6_): δ 32.1 (CH_2_), 38.0 (CH_2_), 118.9 (CH), 120.1 (CH), 120.4 (C), 125.2 (CH), 128.2 (C), 130.9 (CH), 132.9 (CH), 133.0 (CH), 155.7 (C), 159.6 (C), 166.0 (C), 167.6 (C), 172.4 (C). Anal. (C_19_H_15_NO_5_S) calcd: C 61.78; H 4.09; N 3.97; found: C 61.97; H 3.98; N 4.01.

*3-{(5Z)-[5-(3-Benzyloxyphenyl)methylidene]-2,4-dioxothiazolidin-3-yl}propanoic acid* (**3c**). Yield 25% (161.0 mg); m.p. 142–145 °C; ^1^H-NMR (DMSO-*d*_6_): δ 2.60 (t *J* = 7.5 Hz, 2H, CH_2_COOH); 3.86 (t *J* = 7.5 Hz, 2H, NCH_2_); 5.17 (s, 2H, OCH_2_); 7.14–7.20 (m, 2H, arom); 7.24 (s, 1H, arom); 7.34 (m, 1H, arom); 7.40 (m, 2H arom); 7.44–7.48 (m, 3H, arom); 7.88 (s, 1H, methylidene); 12.45 (s, 1H, COOH). ^13^C-NMR (DMSO-*d*_6_): δ 32.0 (CH_2_), 38.0 (CH_2_), 70.0 (CH_2_), 116.7 (CH), 118.0 (C), 122.3 (C), 122.9 (CH), 128.3 (CH), 128.5 (CH), 129.1 (CH), 131.1 (CH), 133.3 (CH), 134.9 (CH), 137.2 (C), 159.3 (C), 165.9 (C), 167.6 (C), 172.4 (C). Anal. (C_20_H_17_NO_5_S) calcd: C 62.65; H 4.47; N 3.65; found: C 62.83; H 4.51; N 3.48.

*3-{(5Z)-[5-(4-Benzyloxyphenyl)methylidene]-2,4-dioxothiazolidin-3-yl}propanoic acid* (**3d**). Yield 61% (392.9 mg); m.p. 195–199 °C; ^1^H-NMR (DMSO-*d*_6_): δ 2.58 (t *J* = 7.5 Hz, 2H, CH_2_COOH); 3.85 (t *J* = 7.5 Hz, 2H, NCH_2_); 5.19 (s, 2H, OCH_2_); 7.18 (m, 2H, arom); 7.34 (m, 1H, arom); 7.40 (m, 2H, arom); 7.46 (m, 2H arom); 7.59 (m, 2H, arom); 7.88 (s, 1H, methylidene); 12.42 (s, 1H, COOH). ^13^C-NMR (DMSO-*d*_6_): δ 32.2 (CH_2_), 38.0 (CH_2_), 70.1 (CH_2_), 116.3 (CH), 118.7 (C), 126.2 (C), 128.4 (CH), 128.6 (CH), 129.1 (CH), 132.8 (CH), 133.4 (CH), 134.9 (CH), 137.0 (C), 160.8 (C), 166.1 (C), 167.7 (C), 172.6 (C). Anal. (C_20_H_17_NO_5_S) calcd: C 62.65; H 4.47; N 3.65; found: C 62.78; H 4.56; N 3.55.

*3-[(5Z)-2,4-Dioxo-{5-[3-(2-phenylethoxy)phenyl]methylidene}thiazolidin-3-yl]propanoic acid* (**3e**). Yield 81% (540.8 mg); m.p. 130–133 °C; ^1^H-NMR (DMSO-*d*_6_): δ 2.60 (t *J* = 7.5 Hz, 2H, CH_2_COOH); 3.06 (t *J* = 6.8 Hz, 2H, CH_2_Ph); 3.86 (t *J* = 7.5 Hz, 2H, NCH_2_); 4.25 (t *J* = 6.8 Hz, 2H, OCH_2_); 7.07 (m, 1H, arom); 7.16–7.24 (m, 3H, arom); 7.3–7.35 (m, 4H, arom); 7.44 (m, 1H arom); 7.89 (s, 1H, methylidene); 12.45 (s, 1H, COOH). ^13^C-NMR (DMSO-*d*_6_): δ 32.0 (CH_2_), 35.4 (CH_2_), 38.0 (CH_2_), 68.9 (CH_2_), 116.6 (CH), 117.7 (C), 122.2 (C), 122.4 (CH), 126.9 (CH), 128.9 (CH), 129.5 (CH), 131.1 (CH), 133.4 (CH), 134.9 (CH), 138.8 (C), 159.4 (C), 165.9 (C), 167.6 (C), 172.4 (C). Anal. (C_21_H_19_NO_5_S) calcd: C 63.46; H 4.82; N 3.52; found: C 63.66; H 4.89; N 3.48

*3-[(5Z)-2,4-Dioxo-{5-[4-(2-phenylethoxy)phenyl]methylidene}thiazolidin-3-yl]propanoic acid* (**3f**). Yield 73% (487.4 mg); m.p. 160–163 °C; ^1^H-NMR (DMSO-*d*_6_): δ 2.59 (t *J* = 7.2 Hz, 2H, CH_2_COOH); 3.06 (t *J* = 6.9 Hz, 2H, CH_2_Ph); 3.85 (t *J* = 7.2 Hz, 2H, NCH_2_); 4.28 (t *J* = 6.9 Hz, 2H, OCH_2_); 7.11 (m, 2H, arom); 7.23 (m, 1H, arom); 7.30–7.34 (m, 4H, arom); 7.57 (m, 2H, arom); 7.87 (s, 1H, methylidene); 12.44 (s, 1H, COOH). ^13^C-NMR (DMSO-*d*_6_): δ 32.1 (CH_2_), 35.3 (CH_2_), 37.9 (CH_2_), 69.0 (CH_2_), 116.0 (CH), 118.5 (C), 126.0 (C), 126.9 (CH), 128.9 (CH), 129.5 (CH), 132.8 (CH), 133.5 (CH), 138.6 (C), 160.9 (C), 166.1 (C), 167.7 (C), 172.4 (C). Anal. (C_21_H_19_NO_5_S) calcd: C 63.46; H 4.82; N 3.52; found: C 63.55; H 4.80; N 3.54.

#### 3.1.3. General Procedure for the Synthesis of 3-(5-arylidene-4-oxo-2-thioxothiazolidin-3-yl)propanoic Acids **4a**–**f**

To a solution of β-alanine (1 g, 12.49 mmol) and NaOH (0.5 g, 12.49 mmol) in H_2_O (35 mL) CS_2_ (2.25 mL, 37.46 mmol) was added dropwise, in such a manner that the temperature of the reaction did not exceed 25 °C and the mixture was stirred at room temperature for 24 h. A solution of sodium bromoacetate, obtained by solubilizing bromoacetic acid (1.73 g, 12.49 mmol) and NaOH (0.5 g, 12.49 mmol) in H_2_O (45 mL), was added and the mixture was stirred at room temperature for a further 24 h. The mixture was acidified with HCl 6M until pH 3–4 and refluxed for 24 h. The mixture was then cooled and poured into H_2_O, providing a pale yellow precipitate. Then, if necessary, pH was adjusted again to 3–4 by the addition of HCl 6M and the solid was filtered off. The aqueous solution was extracted with ethyl acetate; the organic phase was washed with H_2_O, dried with anhydrous Na_2_SO_4_ and evaporated under reduced pressure, providing a further amount of 3-(4-oxo-2-thioxothiazolidin-3-yl)propanoic acid (**7**). The solid residue and the precipitate were collected and washed with ethyl ether to give pure compound **7**. Yield 35% (897.2 mg); mp 159–162 °C; ^1^H-NMR (CDCl_3_): δ 2.77 (t *J* = 6 Hz, 2H, CH_2_COOH), 4.01 (s, 2H, 5-CH_2_) 4.31 (t *J* = 6 Hz, 2H, NCH_2_). Anal. (C_6_H_7_NO_3_S_2_) calcd: C 35.11; H 3.44; N 6.82; found: C 34.96, H 3.34, N 6.92.

A mixture of 3-(4-oxo-2-thioxothiazolidin-3-yl)propanoic acid (**7**) (0.25 g, 1.22 mmol) glacial acetic acid (10 mL), sodium acetate (1.24 g, 9.13 mmol) and the appropriate aldehyde (1.22 mmol) was refluxed for 3–4 h. The mixture was cooled and poured into H_2_O, providing a precipitate which was filtered off, washed with H_2_O and recrystallized from methanol to give pure 3-[(5-arylidene-4-oxo-2-thioxothiazolidin-3-yl)]propanoic acids **4**.

*3-{(5Z)[4-Oxo-5-(3-phenoxyphenyl)methylidene-2-thioxothiazolidin-3-yl]}propanoic acid* (**4a**). Yield 46% (216.3 mg); m.p. 174–175 °C; ^1^H-NMR (DMSO-*d*_6_): δ 2.57 (t *J* = 7.5 Hz, 2H, CH_2_COOH); 4.15 (t *J* = 7.5 Hz, 2H, NCH_2_); 7.01–7.03 (m, 3H, arom); 7.08 (m, 1H, arom); 7.18 (m, 1H, arom); 7.27 (m, 1H, arom); 7.38–7.41 (m, 2H, arom); 7.49 (m, 1H, arom); 7.63 (s, 1H, CH methylidene). ^13^C-NMR (DMSO-*d*_6_): δ 31.9 (CH_2_), 41.1 (NCH_2_), 120.0 (CH), 120.4 (CH), 120.5 (C), 121.8 (CH), 124.3 (CH), 125.5 (CH), 126.5 (CH), 131.4 (CH), 132.4 (CH), 133.3 (CH), 135.7 (C), 156.7 (C), 158.8 (C), 167.7 (C), 173.1 (C), 194.0 (C). Anal. (C_19_H_15_NO_4_S_2_) calcd: C 59.20; H 3.92; N 3.63; found: C 59.06; H 4.01; N 3.60.

*3-{(5Z)[4-Oxo-5-(4-phenoxyphenyl)methylidene-2-thioxothiazolidin-3-yl]}propanoic acid* (**4b**). Yield 46% (216.3 mg); m.p. 218–219 °C; ^1^H-NMR (DMSO-*d*_6_): δ 2.62 (t *J* = 7.5 Hz, 2H, CH_2_COOH); 4.22 (t *J* = 7.5 Hz, 2H, NCH_2_); 7.11 (m, 3H, arom); 7.24 (m, 2H, arom); 7.45 (m, 2H, arom); 7.64 (m, 2H, arom); 7.76 (s, 1H, CH methylidene). ^13^C-NMR (DMSO-*d*_6_): δ 31.7 (CH_2_), 40.8 (NCH_2_), 119.2 (CH), 120.8 (CH), 121.4 (C), 125.7 (CH), 128.4 (CH), 131.2 (C), 133.4 (CH), 133.9 (CH), 155.8 (C), 160.3 (C), 167.6 (C), 172.7 (C), 194.0 (C). Anal. (C_19_H_15_NO_4_S_2_) calcd: C 59.20; H 3.92; N 3.63; found: C 59.25; H 4.00; N 3.57.

*3-{(5Z)-5-[(3-Benzyloxyphenyl)methylidene]-4-oxo-2-thioxothiazolidin-3-yl}propanoic acid* (**4c**). Yield 62% (302.2 mg); m.p. 207–209 °C; ^1^H-NMR (DMSO-*d*_6_): δ 2.62 (t *J* = 7.5 Hz, 2H, CH_2_COOH); 4.21 (t *J* = 7.5 Hz, 2H, NCH_2_); 5.16 (s, 2H, OCH_2_); 7.15–7.19 (m, 3H, arom); 7.33 (m, 1H, arom); 7.39 (m, 2H, arom); 7.44–7.47 (m, 3H, arom); 7.73 (s, 1H, CH methylidene). ^13^C-NMR (DMSO-*d*_6_): δ 31.8 (CH_2_), 41.0 (CH_2_), 70.4 (CH_2_), 117.3 (CH), 119.0 (C), 123.8 (CH), 124.0 (C), 128.7 (CH), 129.1 (CH), 129.6 (CH), 131.8 (CH), 133.9 (CH), 135.2 (CH), 137.5 (C), 159.7 (C), 167.7 (C), 172.9 (C), 194.2 (C). Anal. (C_20_H_17_NO_4_S_2_) calcd: C 60.13; H 4.29; N 3.51; found: C 59.97; H 4.18; N 3.61.

*3-{(5Z)-5-[(4-Benzyloxyphenyl)methylidene]-4-oxo-2-thioxothiazolidin-3-yl}propanoic acid* (**4d**). Yield 49% (238.8 mg); m.p. 170–173 °C; ^1^H-NMR (DMSO-*d*_6_): δ 2.61 (t *J* = 7.5 Hz, 2H, CH_2_COOH); 4.21 (t *J* = 7.5 Hz, 2H, NCH_2_); 5.20 (s, 2H, OCH_2_); 7.19 (m, 2H, arom); 7.34–7.48 (m, 5H, arom); 7.62 (m, 2H, arom); 7.78 (s, 1H, CH methylidene). ^13^C-NMR (DMSO-*d*_6_): δ 31.5 (CH_2_), 40.6 (CH_2_), 70.2 (CH_2_), 116.5 (CH), 119.8 (C), 126.3 (C), 128.4 (CH), 128.6 (CH), 129.1 (CH), 133.5 (CH), 133.7 (CH), 137.0 (C), 161.2 (C), 167.3 (C), 172.4 (C), 193.7 (C). Anal. (C_20_H_17_NO_4_S_2_) calcd: C 60.13; H 4.29; N 3.51; found: C 60.17; H 4.35; N 3.45.

*3-[(5Z)-4-Oxo-{5-[3-(2-phenylethoxy)phenyl]methylidene}-2-thioxothiazolidin-3-yl]propanoic acid* (**4e**). Yield 26% (131.2 mg); m.p. 136–138 °C; ^1^H-NMR (DMSO-*d*_6_): δ 2.63 (t *J* = 7.5 Hz, 2H, CH_2_COOH); 3.05 (t *J*= 6.8 Hz, 2H, CH_2_Ph); 4.20–4.26 (m, 4H, NCH_2_ and OCH_2_); 7.08 (m, 1H, arom); 7.15–7.18 (m, 2H, arom); 7.22 (m, 1H, arom); 7.29–7.34 (m, 4H, arom); 7.45 (m, 1H, arom); 7.76 (s, 1H, CH methylidene). ^13^C-NMR (DMSO-*d*_6_): δ 31.5 (CH_2_), 35.5 (CH_2_), 40.7 (CH_2_), 69.1 (CH_2_), 117.0 (CH), 118.3 (C), 123.2 (C), 123.4 (CH), 127.1 (CH), 129.1 (CH), 129.7 (CH), 131.4 (CH), 133.7 (CH), 135.0 (CH), 138.9 (C), 159.6 (C), 167.3 (C), 172.5 (C), 193.9 (C). Anal. (C_21_H_19_NO_4_S_2_) calcd: C 61.00; H 4.63; N 3.39; found: C 61.18; H 4.74; N 3.25.

*3-[(5Z)-4-Oxo-{5-[4-(2-phenylethoxy)phenyl]methylidene}-2-thioxothiazolidin-3-yl]propanoic acid* (**4f**). Yield 26% (131.2 mg); m.p. 171–174 °C; ^1^H-NMR (DMSO-*d*_6_): δ 2.63 (t *J* = 7.5 Hz, 2H, CH_2_COOH); 3.05 (t *J*= 6.8 Hz, 2H, CH_2_Ph); 4.23 (t *J* = 7.5 Hz, 2H, NCH2); 4.29 (t *J* = 6.8 Hz, 2H, OCH_2_); 7.12 (m, 2H, arom); 7.23 (m, 1H, arom); 7.30–7.34 (m, 4H, arom); 7.59 (m, 2H, arom); 7.77 (s, 1H, CH methylidene); 12.51 (s, 1H, COOH). ^13^C-NMR (DMSO-*d*_6_): δ 31.4 (CH_2_), 35.3 (CH_2_), 40.6 (CH_2_), 69.1 (CH_2_), 116.2 (CH), 119.6 (C), 126.1 (C), 126.9 (CH), 128.9 (CH), 129.5 (CH), 133.5 (CH), 133.8 (CH), 138.6 (C), 161.3 (C), 167.3 (C), 172.3 (C), 193.7 (C). Anal. (C_21_H_19_NO_4_S_2_) calcd: C 61.00; H 4.63; N 3.39; found: C 60.92; H 4.58; N 3.51.

#### 3.1.4. General Procedure for the Synthesis of 4-(5-arylidene-2,4-dioxothiazolidin-3-yl)-2-butenoic Acids **5a**–**e**

A mixture of 5-arylidene-2,4-thiazolidinedione **6** (2.5 mmol), methyl 4-bromocrotonate (0.9 g, 5 mmol) and potassium carbonate (0.69 g, 5 mmol) in anhydrous acetone (50 mL) was refluxed for 24 h. After cooling, the inorganic salts were filtered off and the solvent was evaporated under reduced pressure. The solid residue was washed with H_2_O and recrystallized from ethanol providing pure 4-(5-arylidene-2,4-dioxothiazolidin-3-yl)-2-butenoic acid methyl esters. 

A mixture of the corresponding methyl ester (2.1 mmol), glacial acetic acid (8.5 mL) and HCl 12M (2.1 mL) was refluxed for 2 h. The reaction mixture was poured into H_2_O and the precipitate was filtered off. The crude solid was washed with H_2_O and recrystallized from methanol to provide pure 2-butenoic acids **5**. Chemical-physical and spectroscopic data of compounds **5a**–**c** were reported in [26].

*4-{(5Z)-[5-(1-Naphtylmethylidene)-2,4-dioxothiazolidin-3-yl]}-2-butenoic acid* (**5d**). Yield 26% (220.6 mg); m.p. 170–172 °C; ^1^H-NMR (DMSO-*d*_6_): δ 4.47 (d *J* = 5.0 Hz, 2H, NCH_2_); 5.90 (d *J* = 15.5 Hz, 1H, CHCOOH); 6.83 (dt *J* = 15.5 and 5.0 Hz, 1H, CHCH_2_); 7.62–7.72 (m, 4H, arom); 8.03–8.15 (m, 3H, arom); 8.57 (s, 1H, CH methylidene), 12.52 (s, 1H, COOH). ^13^C-NMR (DMSO-*d*_6_): δ 42.4 (CH_2_), 123.7 (CH), 124.1 (CH), 125.6 (CH), 126.2 (CH), 127.1 (CH), 127.5 (CH), 128.1 (CH), 129.5 (CH), 130.7 (CH), 130.8 (C), 131.5 (C), 133.8 (C), 141.2 (CH), 165.4 (C), 167.0 (C), 167.9 (C), 171.3 (C). Anal. (C_18_H_13_NO_4_S) calcd: C 63.71; H 3.86; N 4.13; found: C 63.59; H 3.76; N 4.22.

*4-{(5Z)-[5-(2-Naphtylmethylidene)-2,4-dioxothiazolidin-3-yl]}-2-butenoic acid* (**5e**). Yield 70% (593.9 mg); m.p. 235–238 °C; ^1^H-NMR (DMSO-*d*_6_): δ 4.45 (d *J* = 5.0 Hz, 2H, NCH_2_); 5.87 (d *J* = 16.0 Hz, 1H, CHCOOH); 6.81 (dt *J* = 16.0 and 5.0 Hz, 1H, CHCH_2_); 7.59–7.65 (m, 2H, arom); 7.71 (m, 1H, arom); 7.97 (m, 1H, arom); 8.04–8.08 (m, 3H, arom); 8.22 (s, 1H, CH methylidene); 12.49 (s, 1H, COOH). ^13^C-NMR (DMSO-*d*_6_): δ 42.4 (CH_2_), 122.1 (C), 123.6 (CH), 126.5 (CH), 127.8 (CH), 128.3 (CH), 128.7 (CH), 129.4 (CH), 129.6 (CH), 131.1 (CH), 131.8 (CH), 133.3 (C), 133.7 (C), 133.9 (C), 141.2 (CH), 165.8 (C), 167.0 (C), 167.7 (C). Anal. (C_18_H_13_NO_4_S) calcd: C 63.71; H 3.86; N 4.133; found: C 63.78; H 3.94; N 4.07.

### 3.2. Enzymatic Assays

#### 3.2.1. AR Enzymatic Assay, Expression and Purification 

The human recombinant AR was expressed in BL21(DE3)pLysS *E. coli* cells and purified to electrophoretic homogeneity as previously described [47]. The purified enzyme (specific activity 5.0 U/mg) was stored at −80 °C in 10 mM sodium phosphate buffer pH 7.0 containing 2 mM dithiothreitol and 30% (*w*/*v*) glycerol. AR activity was determined at 37 °C as previously described [48] evaluating the decrease in absorbance at 340 nm linked to NADPH oxidation. The standard assay mixture contained 0.25 M sodium phosphate buffer pH 6.8, 0.18 mM NADPH, 2.4 M ammonium sulfate, 0.5 mM EDTA and 4.7 mM GAL. One unit of enzyme activity is the amount that catalyzes the conversion of 1 μmol of substrate/min in the above assay conditions. 

#### 3.2.2. Inhibition Studies on AR

The determination of IC_50_ (concentration of compound required to determine a 50% inhibition of enzyme activity) values was performed in the above described assay conditions using 2 mM L-idose as substrate. All compounds tested as aldose reductase inhibitors were dissolved in DMSO and added to the assay mixture containing 8 mU of purified AR. The reaction was started by addition of the substrate. The DMSO concentration in all the assays was kept constant at 0.5% (*v*/*v*) in order to avoid effects on AR activity [49]. 

The IC_50_ values were determined by nonlinear regression analysis using Prism GraphPad 6.0 fitting experimental data to the following equation:

(1) $$\frac{v_{i}}{v_{0}}=\;\frac{Max-Min}{1+{\left(\;\frac{I}{IC_{50}}\right)}^{slope}}\;+Min\;$$

In the equation, *v_i_*/*v*_0_, represents the ratio between the activity measured in the presence of the inhibitor and the activity measured in the absence of inhibitor; *Max* and *Min* represent the expected maximal and minimal value of the activity and were fixed at 1 and zero, respectively. Slope, which describes the steepness of curve in the transition region, was fixed at −1. For each compound, at least five different concentrations of inhibitor in the triplicate assay were analyzed.

The kinetic analysis of compounds **4a**, **4e** and **4f** was performed by measuring reaction rates with different L-idose concentrations s (S) in the absence and in the presence of different inhibitors concentrations. Data were analyzed by Linewever-Burk plots. The apparent dissociation constants *K_i_’* (for the ESI complex) and *K_i_* (for the EI complex) were determined from secondary plots of 1/*V_max_* and K_M_/*V_max_* as a function of the inhibitor concentration, respectively.

In order to evaluate the reversibility of the inhibitory action, 8 mU of purified AR were assayed in the presence of 5 μM of compounds **4a**, **4e** and **4f**. In these conditions less than 15% of the enzyme activity was measured in the presence of 2 mM L-idose as substrate. The mixture was then extensively dialysed on Amicon ultrafiltration membrane (cut off 10 KDa) against 10 mM sodium phosphate buffer, pH 7.0. After dialysis, the enzyme activity was again measured as above and compared to that of a mixture in the absence of inhibitor and treated in the same conditions. 

#### 3.2.3. Enzymatic Assays with PTP1B

PTP1B activity was determined as follow. An aliquot of human recombinant PTP1B was diluted in the assay buffer containing 0.075 M β-β-dimethylglutarate pH 7.0, 1 mM EDTA, 0.1 mM DTT and p-nitrophenyl phosphate (*p*NPP) as substrate. After an appropriate interval time, the reaction was stopped diluting assay solution with 2 mL of KOH 0.1 M. The amount of *p*-nitrophenol released was determined measuring the absorbance of the solution at 400 nm using a spectrophotometer and a 1-cm optical pathlength (ε_mM_ of *p*-nitrophenol is 18).

The IC_50_ value was determined measuring the pNPP hydrolysis rate in the presence of increasing inhibitor concentration. For each inhibitor, 15–16 different concentrations were used. All tests were carried out in triplicate and data obtained were normalized respect to control sample. Experimental points were fitted using Equation (1), shows in the Section 3.2.2. 

#### 3.2.4. Reversibility Assay (Dilution Test) with PTP1B

An aliquot of the enzyme was incubated for 60 min at 37 °C in the presence of saturating amount of PTP1B inhibitor. After this time, an aliquot of the enzyme was withdrawn and diluted 400 folds in the assay buffer containing 2.5 mM of *p*NPP. After 15 min, the reaction was stopped by adding 1 mL of KOH 0.2 M. The amount of *p*NP released was calculating measuring the absorbance at 400 nm of solutions using a spectrophotometer. Data obtained were normalized respect to control tests, which was carried out diluting the enzyme with DMSO, the solvent used to dissolve all compounds. Data reported in the figure represent the mean value +/− S.E.M. (*n* = 3).

#### 3.2.5. Evaluation of Action Mechanism of Inhibitors

Kinetic analyses were carried out to dissect action mechanism of compound **4e** and **4f**. Briefly, the main kinetic parameters, Km and Vmax, were calculated measuring the initial hydrolysis rate of PTP1B in the presence of increasing pNPP concentration (seven different concentrations in the 0.5–25 mM range). All assays were carried out in duplicate. Data obtained were fitted using the Michaelis-Menten equation. To evaluate the action mechanism of inhibitors, we studied the dependence of Km and Vmax from inhibitors concentration, using three different inhibitor concentrations, at least. Data obtained were then analysed by double reciprocal plot (Lineweaver-Burk plot). 

### 3.3. Ex Vivo Assay

#### 3.3.1. Cell Cultures

Human liver cells (HepG2) and murine myoblasts (C2C12) were purchased by Sigma-Aldrich, which obtained them from the European Collection of Authenticated Cell Cultures (ECACC). HepG2 and C2C12 cells were grown in Dulbecco’s Modified Eagle’s medium (DMEM) supplemented with 10% foetal bovine serum (FBS), 2 mM glutamine, 100 IU/mL penicillin, 100 μg/mL streptomycin (Sigma-Aldrich, St. Louis, MO, USA), in a humidified atmosphere and with 5% CO_2_ at 37 °C. Differentiation of C2C12 was induced incubating murine myoblasts in DMEM supplemented with 2% horse serum. C2C12 cells were plated and grown until they reach 70% confluence. Then, cells were incubated in the presence of 2% horse serum for 96 h. Every 48 h, the medium was replaced with fresh medium. After 96 h, the degree of differentiation was evaluated analysing cells with a contrast phase microscope.

#### 3.3.2. Cell Viability Assay

HepG2 and C2C12 cells were incubated in the presence of increasing concentrations of compounds **4e** and **4f** for 48 h at 37 °C. After this time, cells were washed with PBS and then incubated for 1 h with 0.5 mg/mL of MTT dissolved in complete medium. Then cells were washed and lysed with 300 μL of DMSO. The absorbance of solutions were evaluated using a microplate readers at 595 nm. All data were normalized respect to control experiment. Data showed in the figures represent the mean values ± S.E.M. (*n* = 4).

#### 3.3.3. Evaluation of Insulin Mimetic/Sensibilizing Activity of Inhibitors

HepG2 and differentiated C2C12 cells were starved for 24 h and then incubated in the presence of compound **4f** (25 μM, final concentration) for 90 min. After this time, cells were washed with PBS and then stimulated or not with 10 nM insulin for 30 min. Then, cells were lysed and cellular proteins separated on a 12% SDS-PAGE. After electrophoresis, cellular proteins were transferred on a PVDF membrane by western blot technique and then probed against antibodies able to specifically recognize insulin receptor (I2033 Sigma Aldrich) or phosphorylated form of IR (MABS65, Sigma-Aldrich). Sample loading was evaluated probing PVDF membrane with anti-actin antibodies (ABT1485 Sigma Aldrich). 

### 3.4. Molecular Docking

Crystal structure selection and preparation for PTP1B (PDB 1Q6T [50]) and the AR-idose complex (based on PDB 3V36 [51]) was carried out as described in our previous study [5]. Docking studies were performed into the catalytic site of PTP1B and AR, the AR-idose complex and our previously described allosteric binding pocket of PTP1B [5] with GOLD (version 5.2) [52], generating 25 docking poses per ligand with standard settings using the scoring function CHEMPLP. In order to select a docking pose, the 25 docking poses were clustered based on LigandScouts [53,54] pharmacophore RDF-Code (radial distribution function) similarity. Finally, all selected docking poses have been energy minimized in Ligand Scout with the MMFF94 Force Field [55]. 3D protein ligand depictions were rendered with MOE (version 2019) [56] and 3D pharmacophores were developed using Ligand Scout.

#### Virtual Screening

Shape-based virtual screening was performed with ROCS (OpenEye, Scientific Software, Santa Fe, NM, USA) [57] using default options. Docking of virtual screening hits was carried out with the software package GOLD (version 5.2) [52] into the catalytic centre of PTP1B (PDB 1Q6T [50]), generating 10 docking poses per ligand with standard settings as described above. The hit compound 3-{[5-(4-benzyloxyphenyl)methylidene]-2,4-dioxothiazolidin-3-yl}propanoic acid was identified by using the co-crystallized ligand (4-bromo-3-(carboxymethoxy)-5-[3-(cyclohexylamino)phenyl]thiophene-2-carboxylic acid) from the PTP1B crystal structure 2QBS [58] as the template for the ROCS screening. The compound was found screening the vendor space of Enamine HTS Collection (as of 06/2014).

## 4. Conclusions

The appreciable in vitro activity of compounds **4a**, **4e** and **4f** as well as **5a** and **5d** as dual AR/PTP1B inhibitors provided further insights into the possibility to develop 4-thiazolidinone derivatives as new potential multitarget antidiabetic candidates. Taking into account that the partial inhibition of selected targets, in particular enzymes, may be therapeutically more effective than the total control of a single target [2,3], these dual inhibitors, along with previous analogues **1e** and **2e**, appear to be promising as lead compounds, whose inhibitory activity towards both target enzymes might be further improved. In particular, compound **4f** exhibited the most interesting profile, with balanced AR/PTP1B inhibitory effectiveness at low micromolar concentrations, along with promising insulin-sensitizing activity in cultured cells. The IC_50_ values of **4f** against both human PTP1B and human AR resulted in being in the low micromolar range, whereas those of the corresponding acetic acid analogue of series **2** differ of two orders of magnitude [5]. Therefore, although the elongation of the carboxylic chain on N-3 of the thiazolidinone scaffold produced a decrease in AR inhibitory effectiveness, this structural feature was responsible for a gain in potency against PTP1B, thus providing a more balanced inhibition profile towards the two target enzymes. 

The SARs that emerged from our enzyme inhibition data, also considering our previous findings, highlighted that the AR inhibitory potency of the investigated 4-thiazolidinones is mainly influenced by the nature of the substituents in positions 2 and 3 of the heterocyclic scaffold and only in a lesser extent by the nature of the 5-arylidene moiety. On the other hand, this latter structural portion appeared to be critical to achieving good PTP1B inhibition, considering that a more extended 5-arylidene portion, in particular a (2-phenylethoxy)benzylidene moiety, showed to be more beneficial. In addition, a thiocarbonyl group in position 2 of the thiazolidinone scaffold generally proved to be advantageous to achieve gain in potency towards both target enzymes. The elongation of the carboxylic chain on N-3 in several cases improved inhibitory effectiveness towards PTP1B; propanoic acid derivatives **4a**, **4e** and **4f** as well as 2-butenoic acid derivatives **5a** and **5d** exhibited good capability to inhibit both human PTP1B and AR. The data reported here allowed us to extend the SARs of this class of DMLs, which were rationalized by in silico docking experiments into both target enzymes. Therefore, these findings can be of interest for the design of new 4-thiazolidinone derivatives endowed with improved pharmacological profiles. 

## Figures and Tables

**Table 1:**
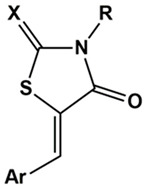
Inhibitory activities of compounds **3**–**5** against human AR and human PTP1B, expressed as IC_50_^a^.

	X	R	Ar	AR IC_50_ (µM)	PTP1B IC_50_ (µM)
**3a**	O	(CH_2_)_2_COOH	3-OC_6_H_5_-C_6_H_4_	11.9 ± 0.9	79% at 50 µM
**3b**	O	(CH_2_)_2_COOH	4-OC_6_H_5_-C_6_H_4_	43.8 ± 7.1	56% at 50 µM
**3c**	O	(CH_2_)_2_COOH	3-OCH_2_C_6_H_5_-C_6_H_4_	14.3 ± 1.0	76% at 50 µM
**3d**	O	(CH_2_)_2_COOH	4-OCH_2_C_6_H_5_-C_6_H_4_	35.7 ± 3.0	77% at 50 µM
**3e**	O	(CH_2_)_2_COOH	3-OCH_2_CH_2_C_6_H_5_-C_6_H_4_	27.9 ± 3.1	64% at 50 µM
**3f**	O	(CH_2_)_2_COOH	4-OCH_2_CH_2_C_6_H_5_-C_6_H_4_	50.2 ± 4.6	46% at 50 µM
**4a**	S	(CH_2_)_2_COOH	3-OC_6_H_5_-C_6_H_4_	2.2 ± 0.1	34.1 ± 0.5
**4b**	S	(CH_2_)_2_COOH	4-OC_6_H_5_-C_6_H_4_	7.6 ± 0.6	29.5 ± 0.4
**4c**	S	(CH_2_)_2_COOH	3-OCH_2_C_6_H_5_-C_6_H_4_	3.8 ± 0.1	42.8 ± 0.7
**4d**	S	(CH_2_)_2_COOH	4-OCH_2_C_6_H_5_-C_6_H_4_	8.4 ± 0.7	34.9 ± 0.7
**4e**	S	(CH_2_)_2_COOH	3-OCH_2_CH_2_C_6_H_5_-C_6_H_4_	2.3 ± 0.1	55.5 ± 0.8
**4f**	S	(CH_2_)_2_COOH	4-OCH_2_CH_2_C_6_H_5_-C_6_H_4_	5.3 ± 0.4	12.7 ± 0.3
**5a**	O	CH_2_CH=CHCOOH	3-OC_6_H_5_-C_6_H_4_	3.9 ± 0.2	42.1 ± 0.3
**5b**	O	CH_2_CH=CHCOOH	4-OC_6_H_5_-C_6_H_4_	84% at 10 µM	39.7 ± 0.1
**5c**	O	CH_2_CH=CHCOOH	4-C_6_H_5_-C_6_H_4_	88% at 5 µM	34.8 ± 0.5
**5d**	O	CH_2_CH=CHCOOH	1-naphthyl	3.7 ± 0.2	40.3 ± 0.5
**5e**	O	CH_2_CH=CHCOOH	2-naphthyl	86% at 10 µM	37.1 ± 0.4
Epalrestat				0.102 ± 0.005	
Vanadate					0.4 ± 0.01

^a^ IC_50_ (µM) or % enzyme residual activity at the indicated concentration. Values are expressed as the mean ± S.E.M (see methods for details).

**Table 2:** Apparent dissociation constants for selected compounds ^a^.

	AR		PTP1B	
Inhibitor	*K_i_* (µM)	*K′_i_* (µM)	*K_i_* (µM)	*K′_i_* (µM)
**4a**	5.0 ± 0.57	1.4 ± 0.022	10.6 ± 1.7	68.9 ± 1.5
**4e**	>8	0.66 ± 0.30	2.2 ± 0.3	>12
**4f**	>30	2.9 ± 0.12	0.9 ± 0.1	2.3 ± 0.1

^a^*K_i_* and *K’_i_* refer to the apparent dissociation constants for the ES and EIS complex, respectively.

## Data Availability

The data presented in this study are available in this article and relative supplementary material.

## Supplementary Materials

The supplementary materials are available online.

## Sample Availability

Samples of the compounds are not available from the authors.
