# Supplementary material for: In Search for Multi-Target Ligands as Potential Agents for Diabetes Mellitus and Its Complications—A Structure-Activity Relationship Study on Inhibitors of Aldose Reductase and Protein Tyrosine Phosphatase 1B
Source: Molecules. 2021 Jan 10;26(2):330. doi: 10.3390/molecules26020330 (PMC7828111; doi:10.3390/molecules26020330)
Supplement: Supplementary file 1 [file molecules-26-00330-s001.pdf]

SUPPLEMENTARY MATERIAL

In search for multi-target ligands as potential agents for diabetes mellitus and its complications: A structure-activity relationship study on inhibitors of aldose reductase and protein tyrosine phosphatase 1B.

Rosaria Ottanà,[a] Paolo Paoli,[b] Mario Cappiello,[c] Trung Ngoc Nguyen,[d] Ilenia Adornato,[a] Antonella Del Corso,[c] Massimo Genovese,[b] Ilaria Nesi,[b] Roberta Moschini,[c] Alexandra Naß,[d] Gerhard Wolber,[d] Rosanna Maccari [a]*

Department of Chemical, Biological, Pharmaceutical and Environmental Sciences, University of Messina, Viale Palatucci, Polo Universitario Annunziata, 98168 Messina, Italy

Department of Biology, Biochemistry Unit, University of Pisa, Via S. Zeno, 51, 56123 Pisa, Italy

Department of Scienze Biomediche Sperimentali e Cliniche, Sezione di Scienze Biochimiche, University of Firenze, Viale Morgagni 50, 50134 Firenze, Italy

Molecular Design Lab, Institute of Pharmacy, Freie Universität Berlin, Königin-Luisestr. 2+4, 14195 Berlin, Germany.

Figures S1-S28. ^{1}H NMR and ^{13}C NMR spectra of representative compounds **3a-f, 4a-f, 5d, 5e.**

Figure S29. Reversibility assay.

Figures S30-S32. Kinetic characterization of compound **4a, 4e, 4f** as AR inhibitors.

Figures S33-S35. Kinetic characterization of compound **4a, 4e, 4f** as PTP1B inhibitors.

Table S1 – Calculated parameters of compounds **3a-f, 4a-f, 5a-e.**

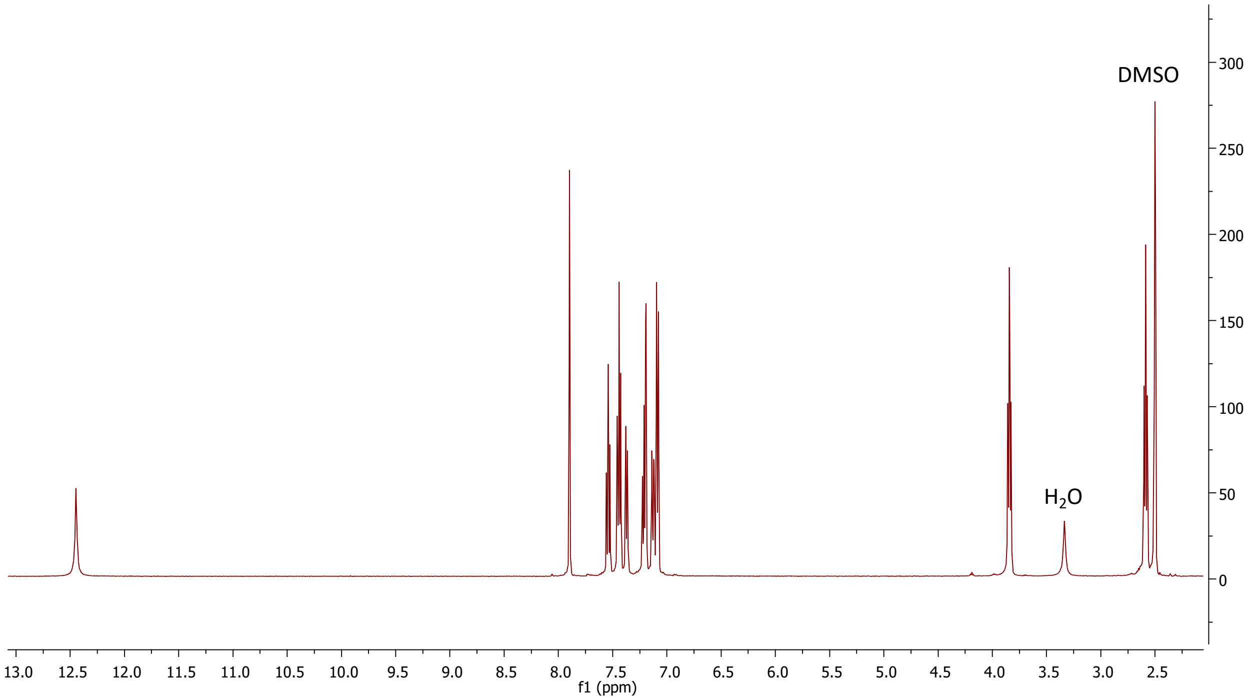

Figure S1 - ^{1}H-NMR spectrum of compound **3a** (500 MHz, DMSO-d_6)

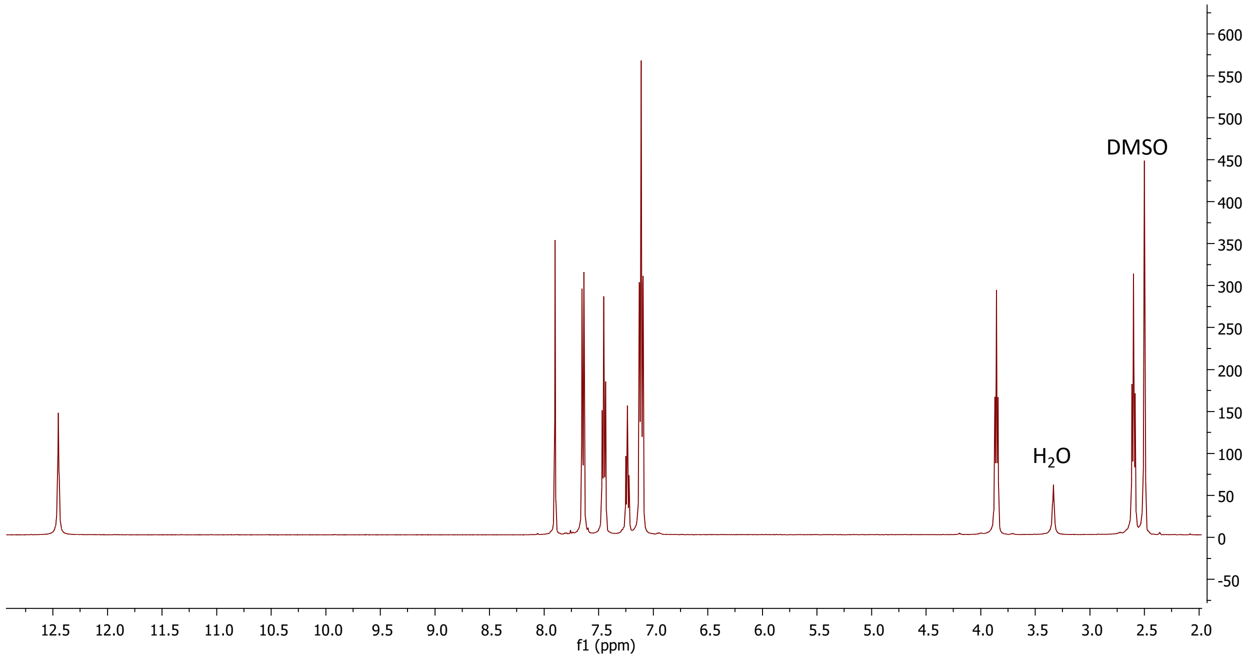

Figure S2 - ^{1}H-NMR spectrum of compound **3b** (500 MHz, DMSO-d_6)

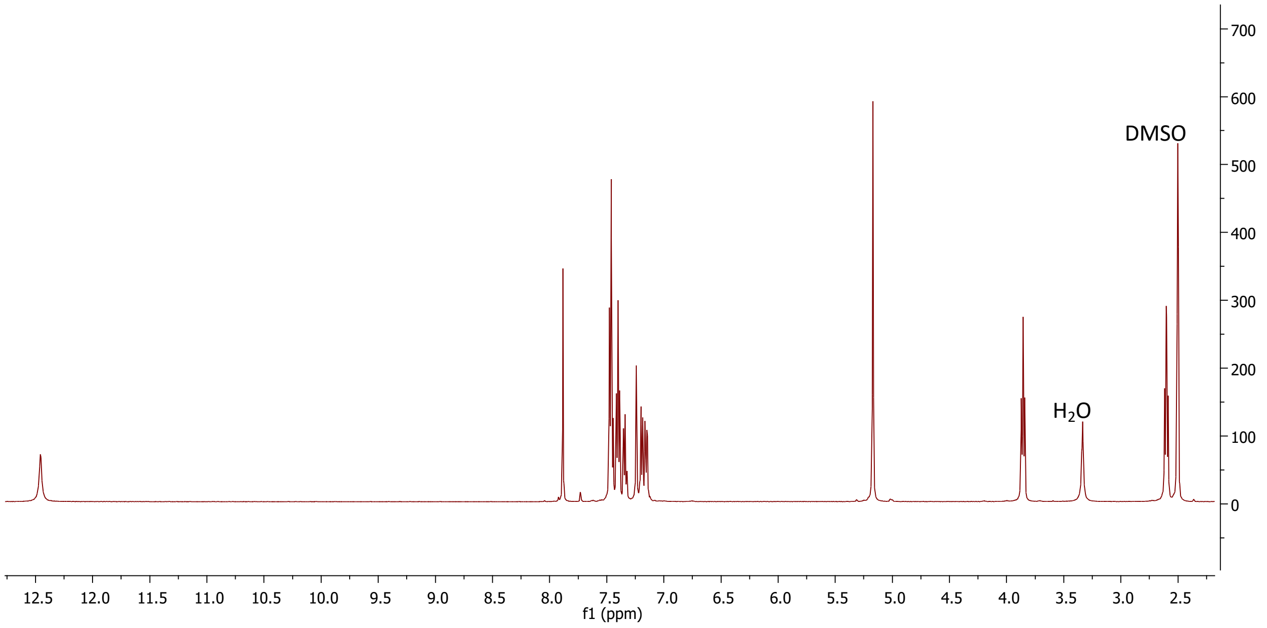

Figure S3 - ^{1}H-NMR spectrum of compound **3c** (500 MHz, DMSO-d_6)

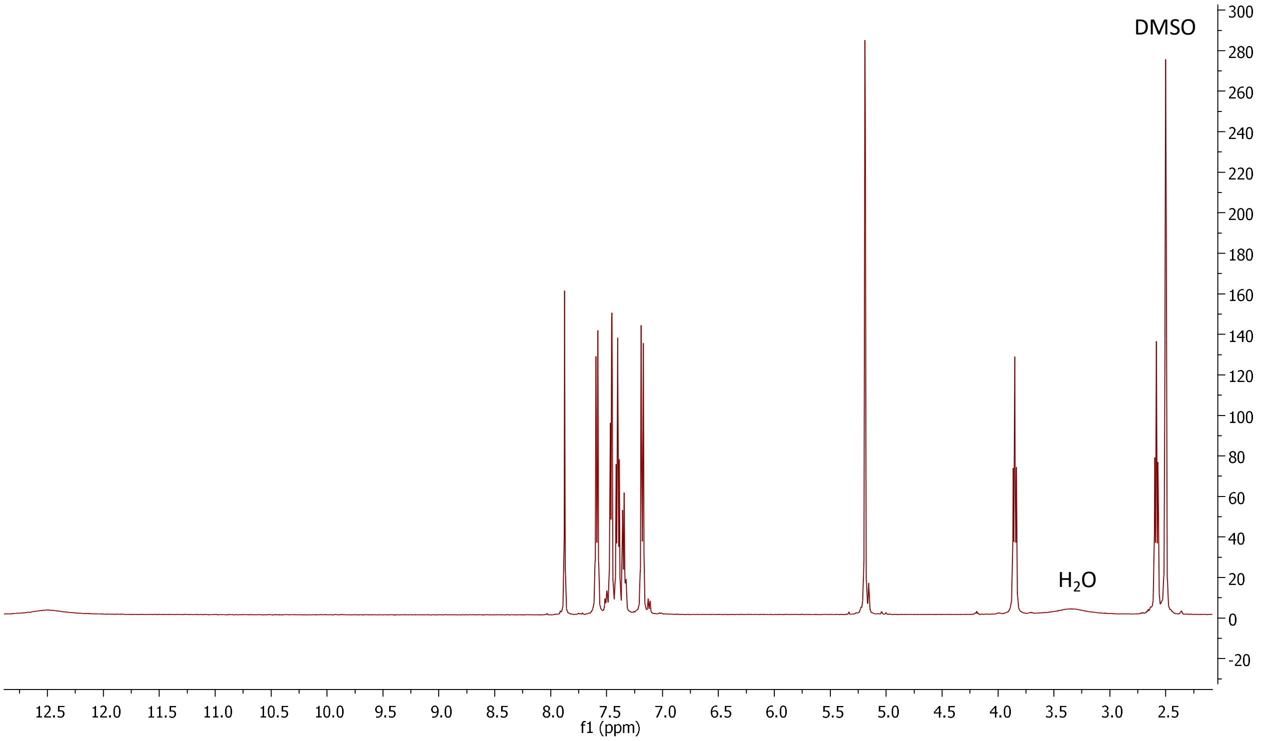

Figure S4 - ^{1}H-NMR spectrum of compound **3d** (500 MHz, DMSO-d_6)

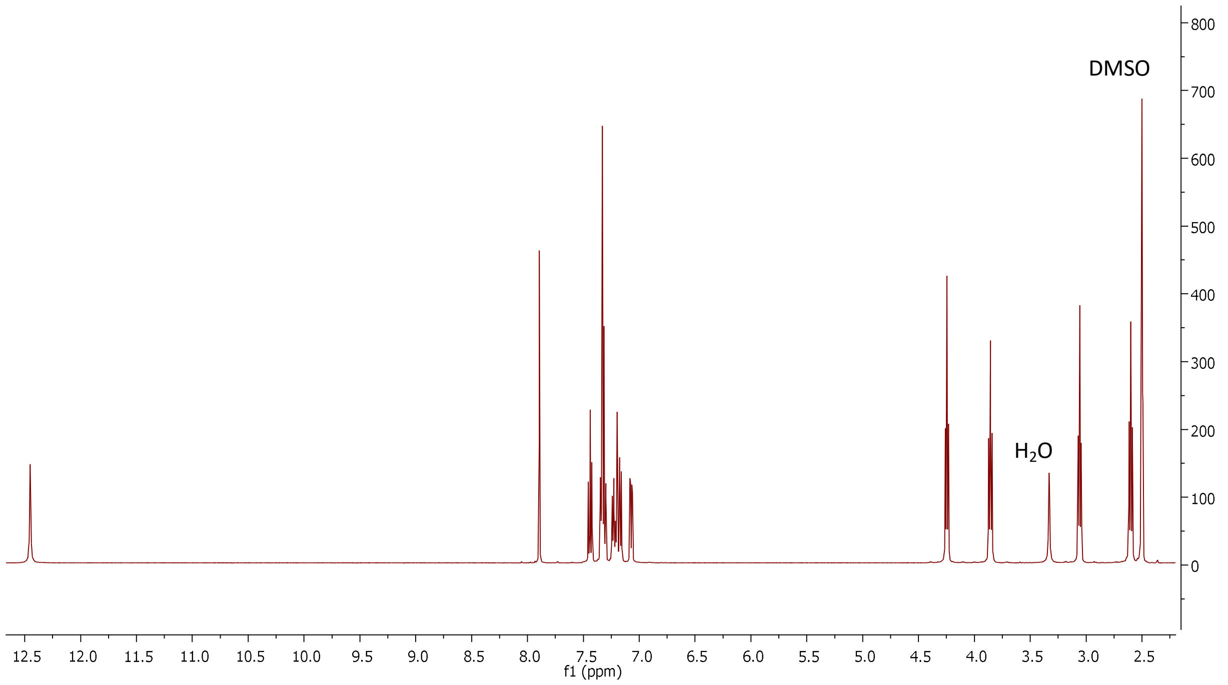

Figure S5 - ^{1}H-NMR spectrum of compound **3e** (500 MHz, DMSO-d_6)

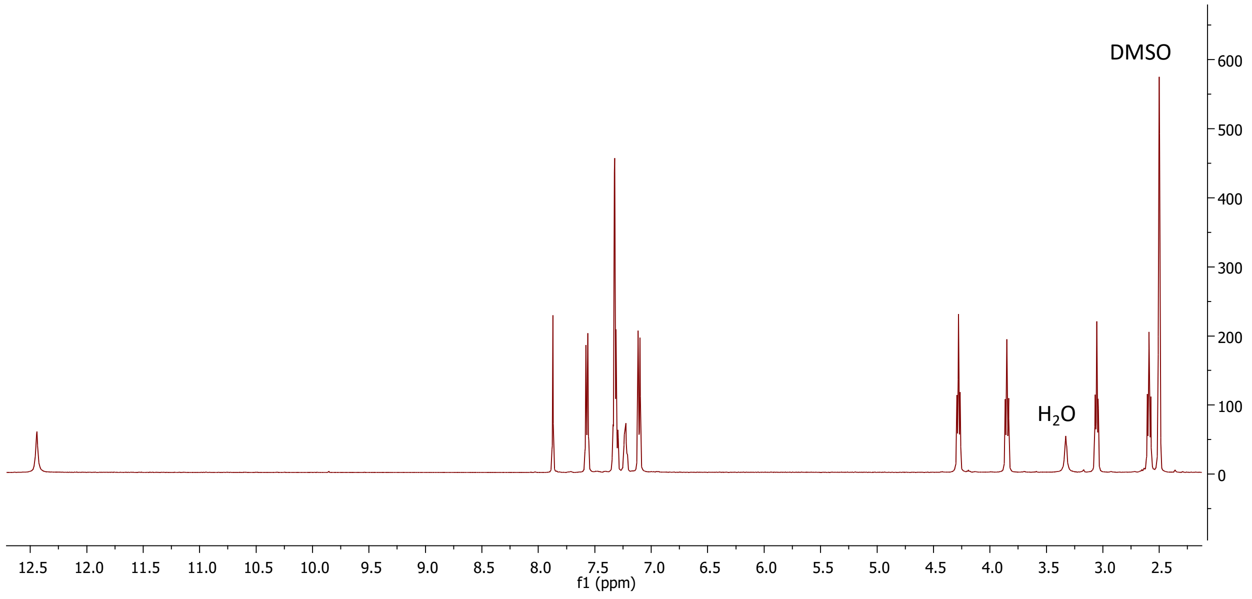

Figure S6 - ^{1}H-NMR spectrum of compound **3f** (500 MHz, DMSO-d_6)

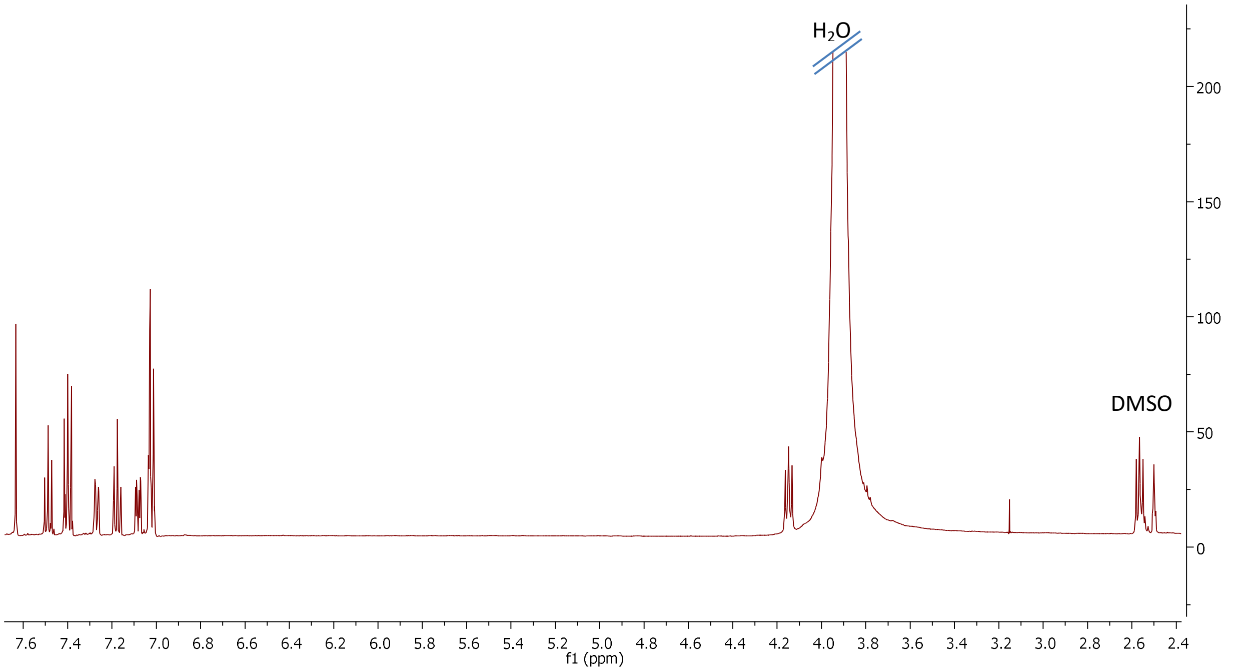

Figure S7 - ^{1}H-NMR spectrum of compound **4a** (500 MHz, DMSO-d_6)

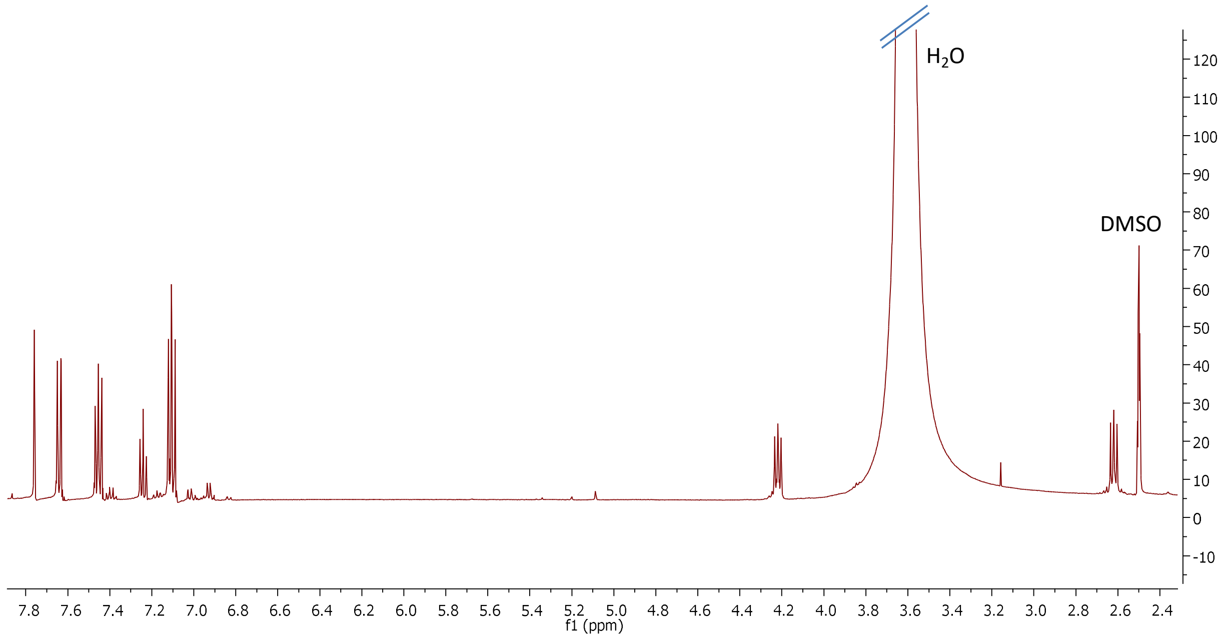

Figure S8 - ^{1}H-NMR spectrum of compound **4b** (500 MHz, DMSO-d_6)

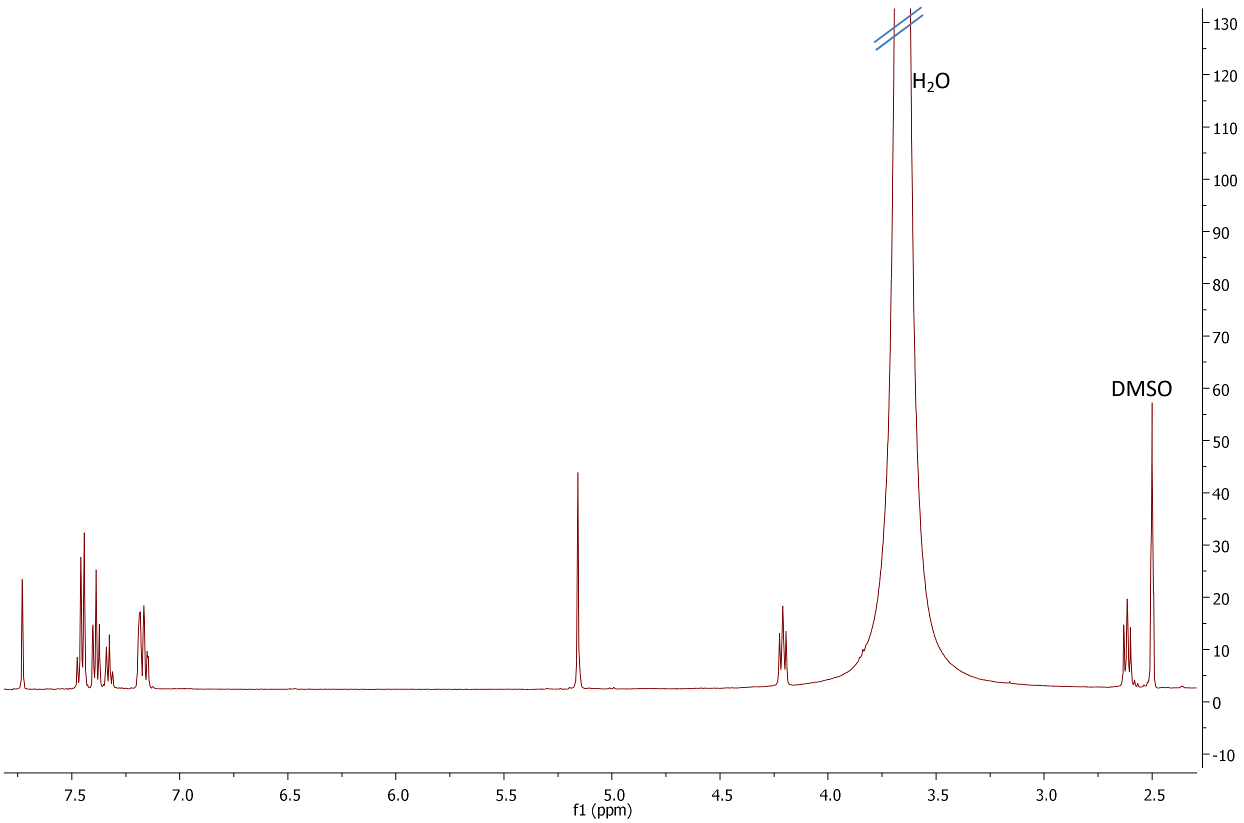

Figure S9 - ^{1}H-NMR spectrum of compound **4c** (500 MHz, DMSO-d_6)

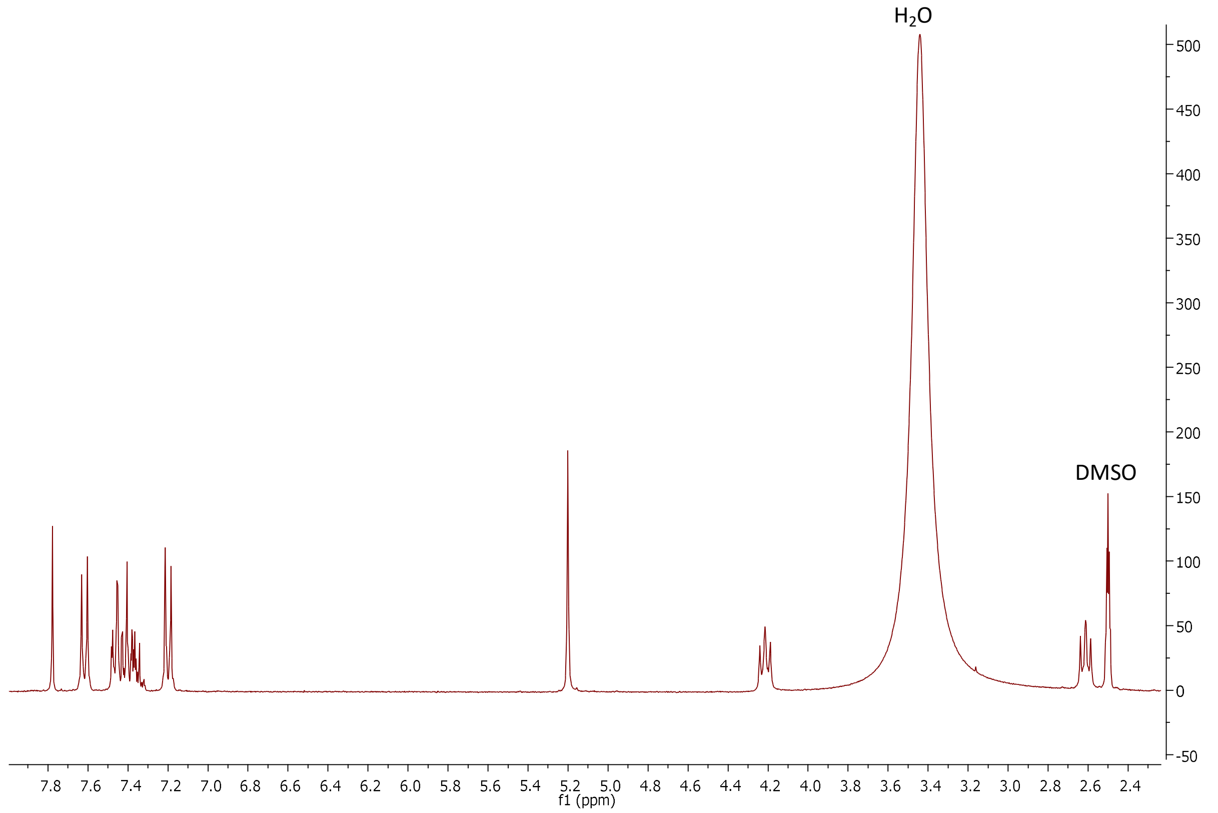

Figure S10 - ^{1}H-NMR spectrum of compound **4d** (500 MHz, DMSO-d_6)

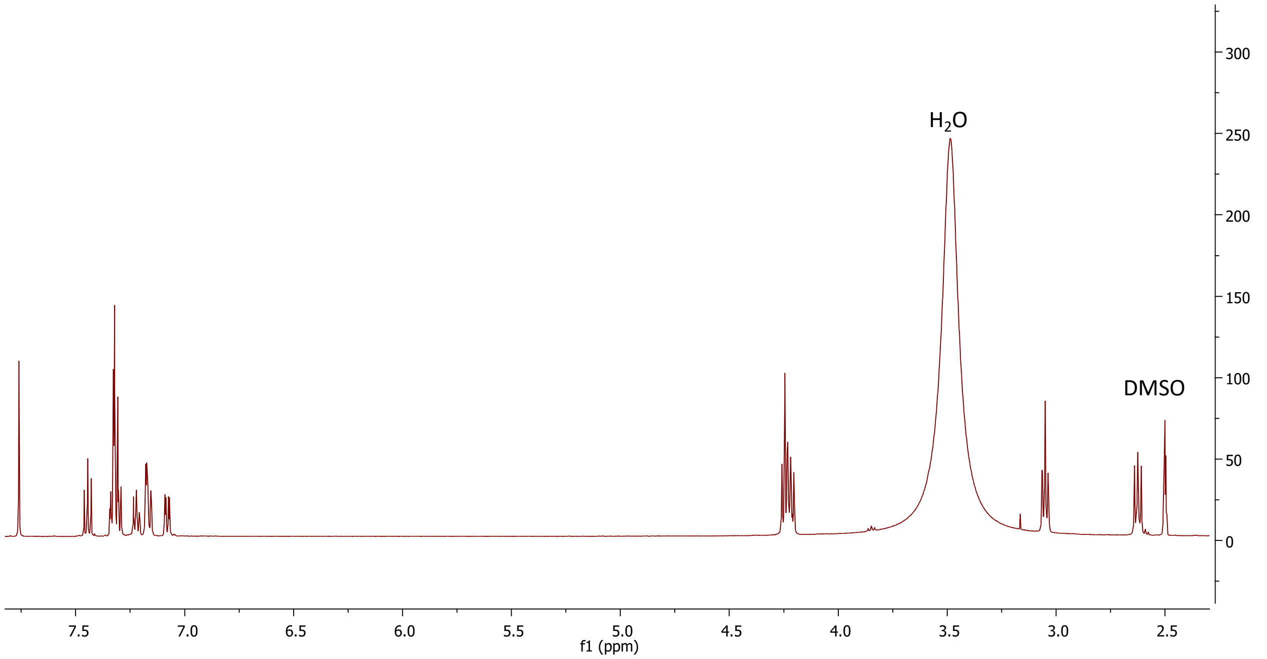

Figure S11 - ^{1}H-NMR spectrum of compound **4e** (500 MHz, DMSO-d_6)

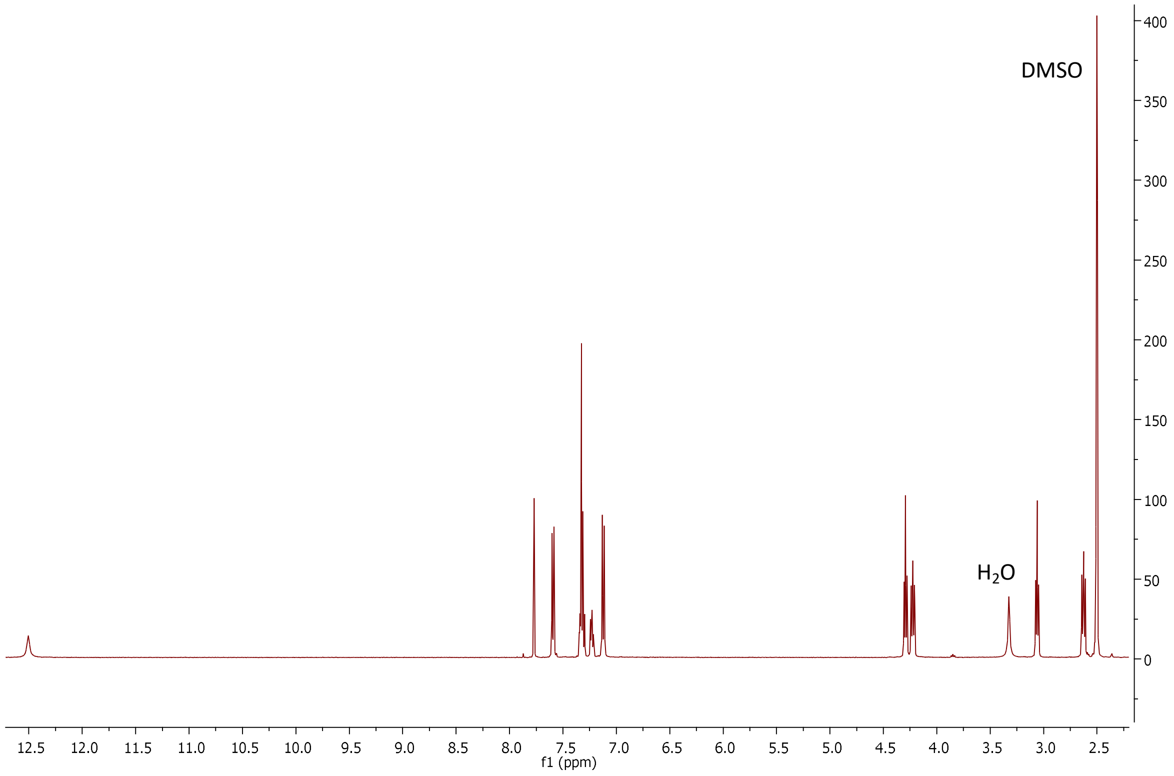

Figure S12 - ^{1}H-NMR spectrum of compound **4f** (500 MHz, DMSO-d_6)

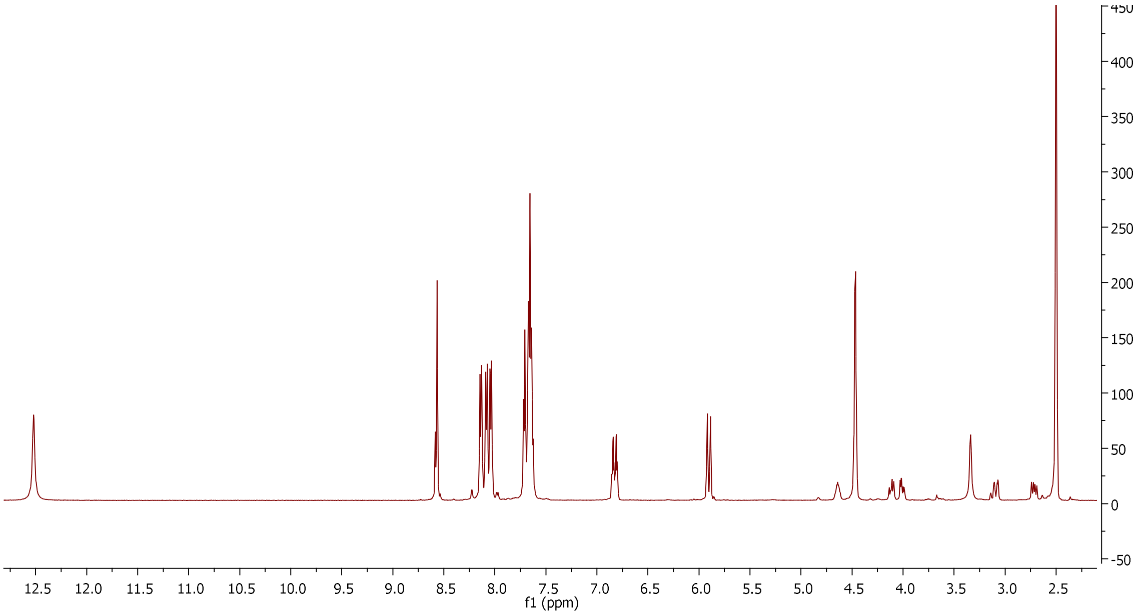

Figure S13 - ^{1}H-NMR spectrum of compound **5d** (500 MHz, DMSO-d_6)

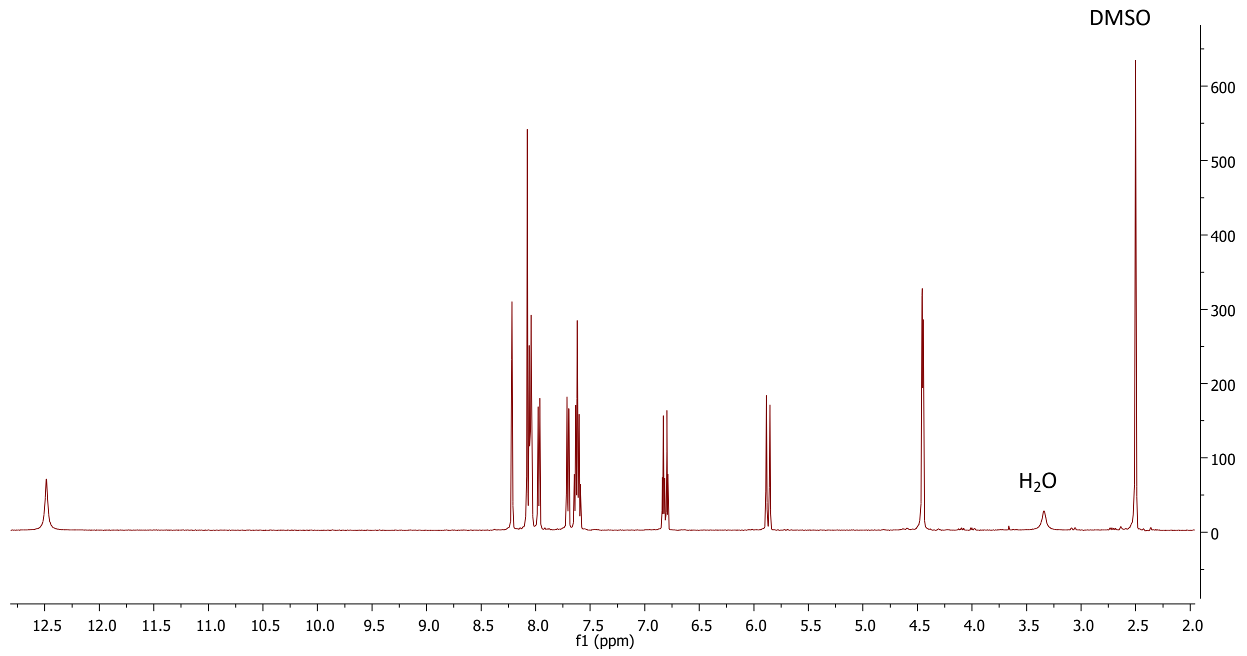

Figure S14 - ^{1}H-NMR spectrum of compound **5e** (500 MHz, DMSO-d_6)

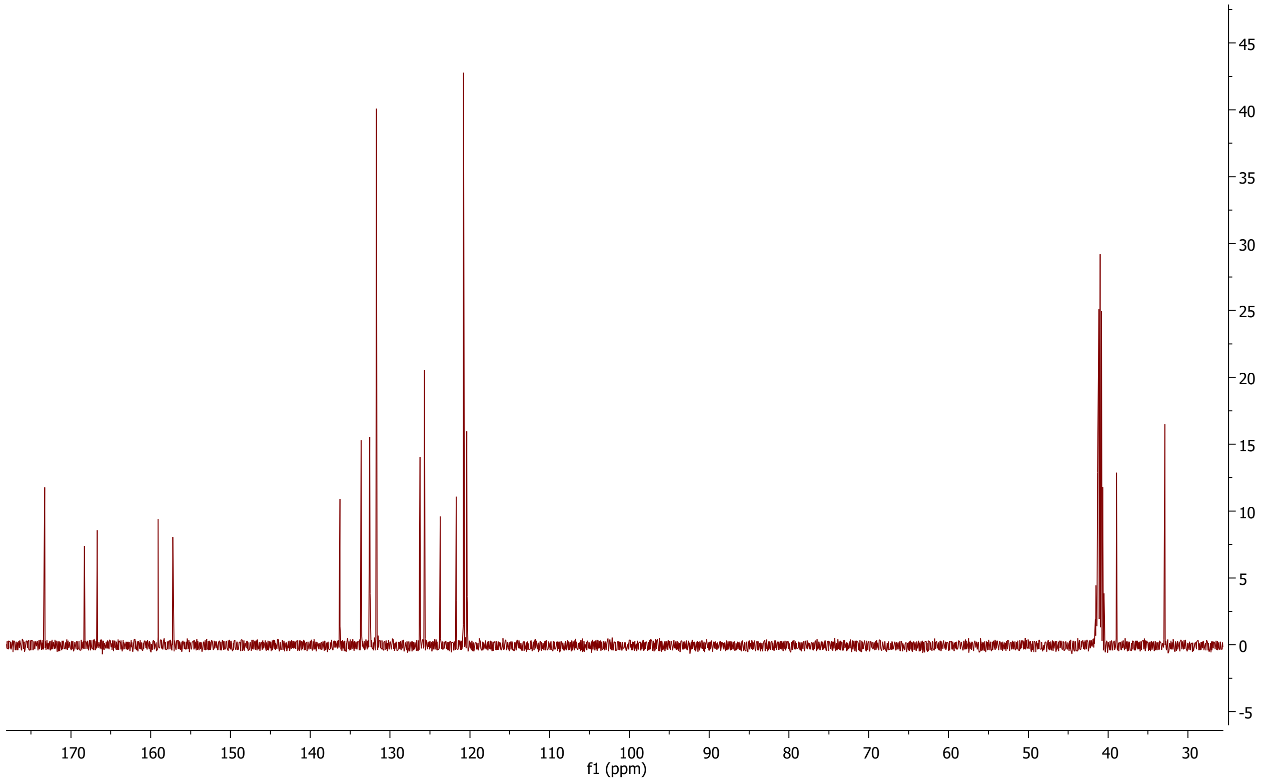

Figure S15 - ^{13}C-NMR spectrum of compound **3a** (125.73 MHz, DMSO-d_6)

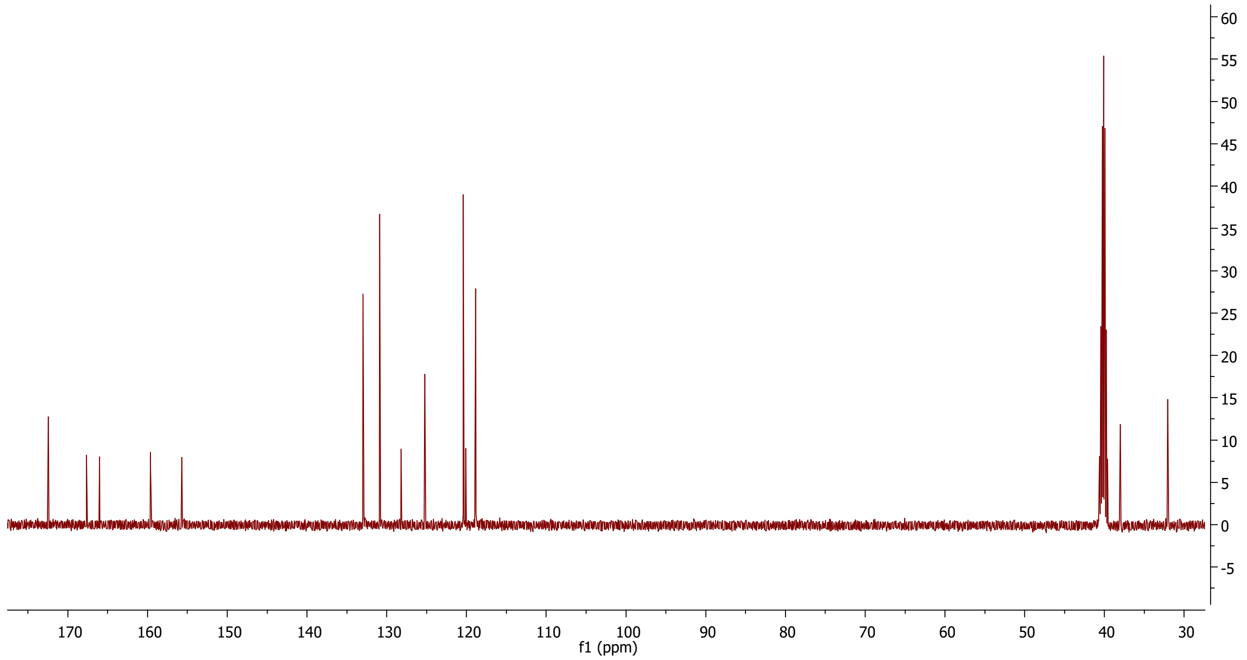

Figure S16 - ^{13}C-NMR spectrum of compound **3b** (125.73 MHz, DMSO-d_6)

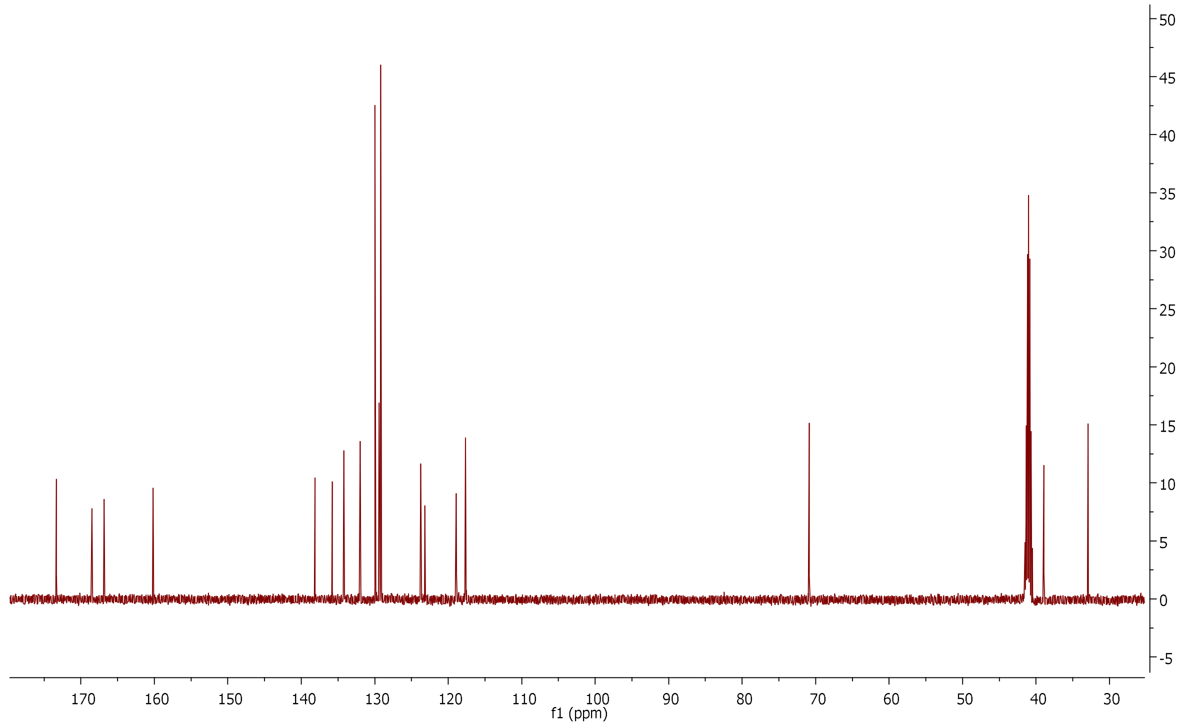

Figure S17 - ^{13}C-NMR spectrum of compound **3c** (125.73 MHz, DMSO-d_6)

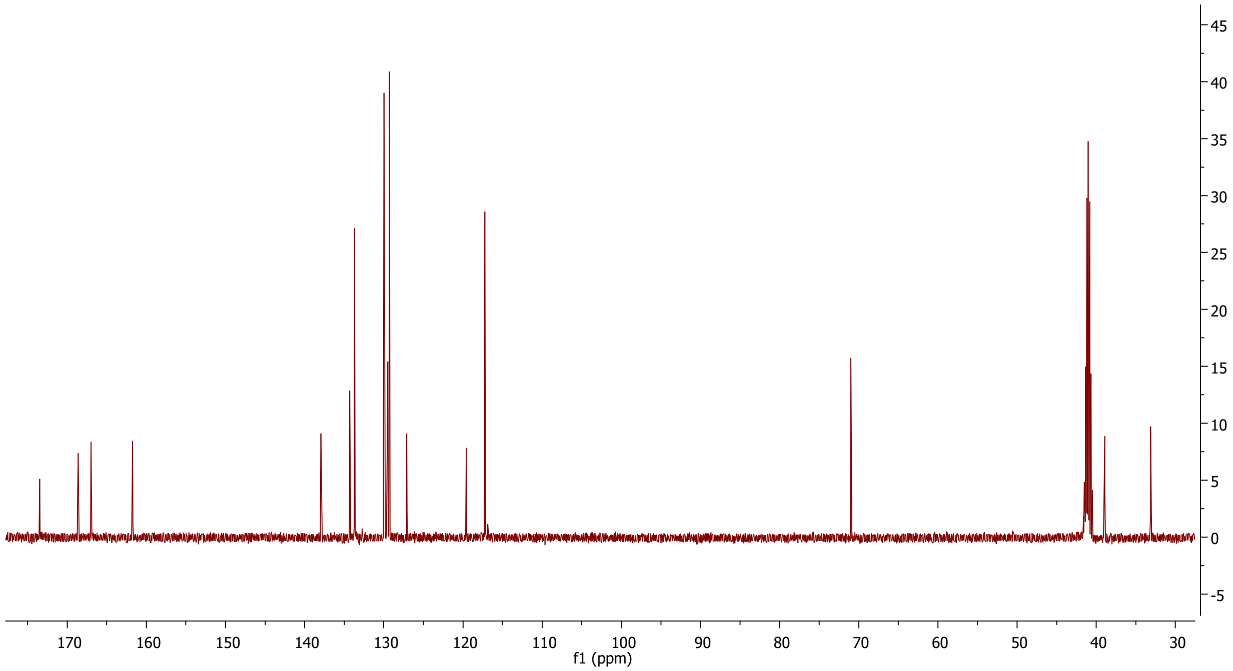

Figure S18^{13}C-NMR spectrum of compound **3d** (125.73 MHz, DMSO-d_6)

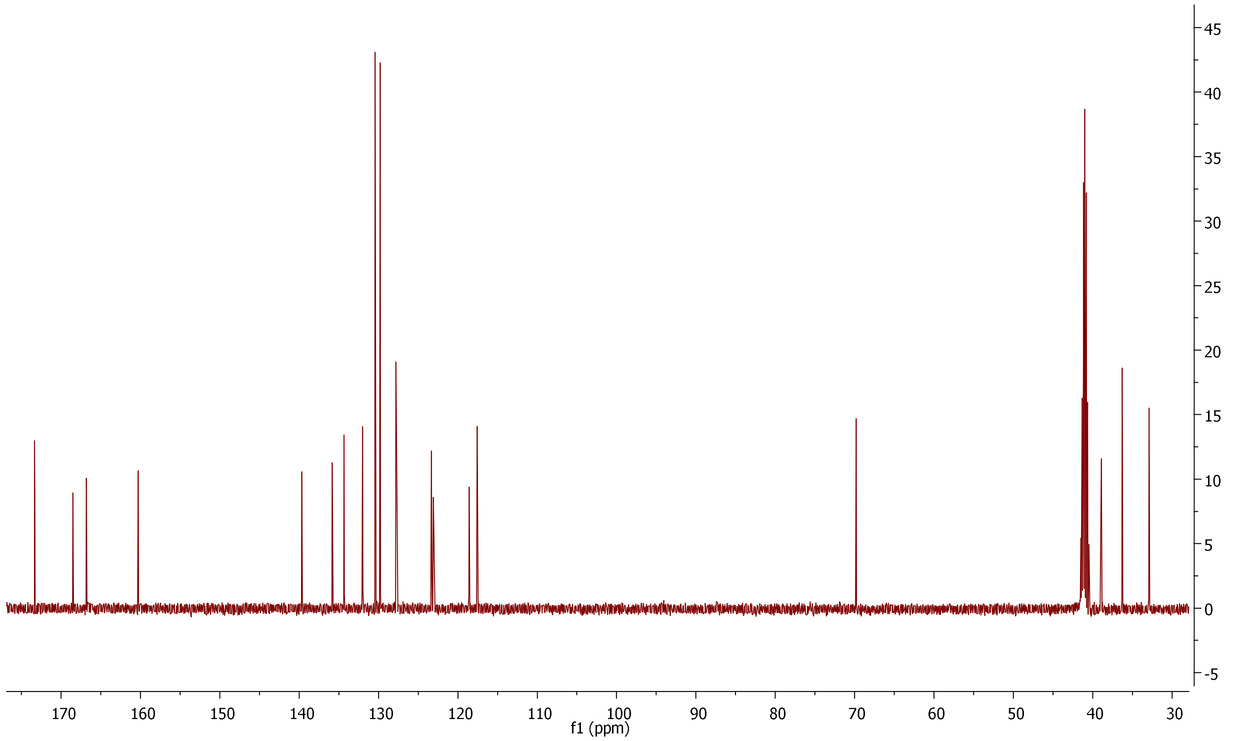

Figure S19 - ^{13}C-NMR spectrum of compound **3e** (125.73 MHz, DMSO-d_6)

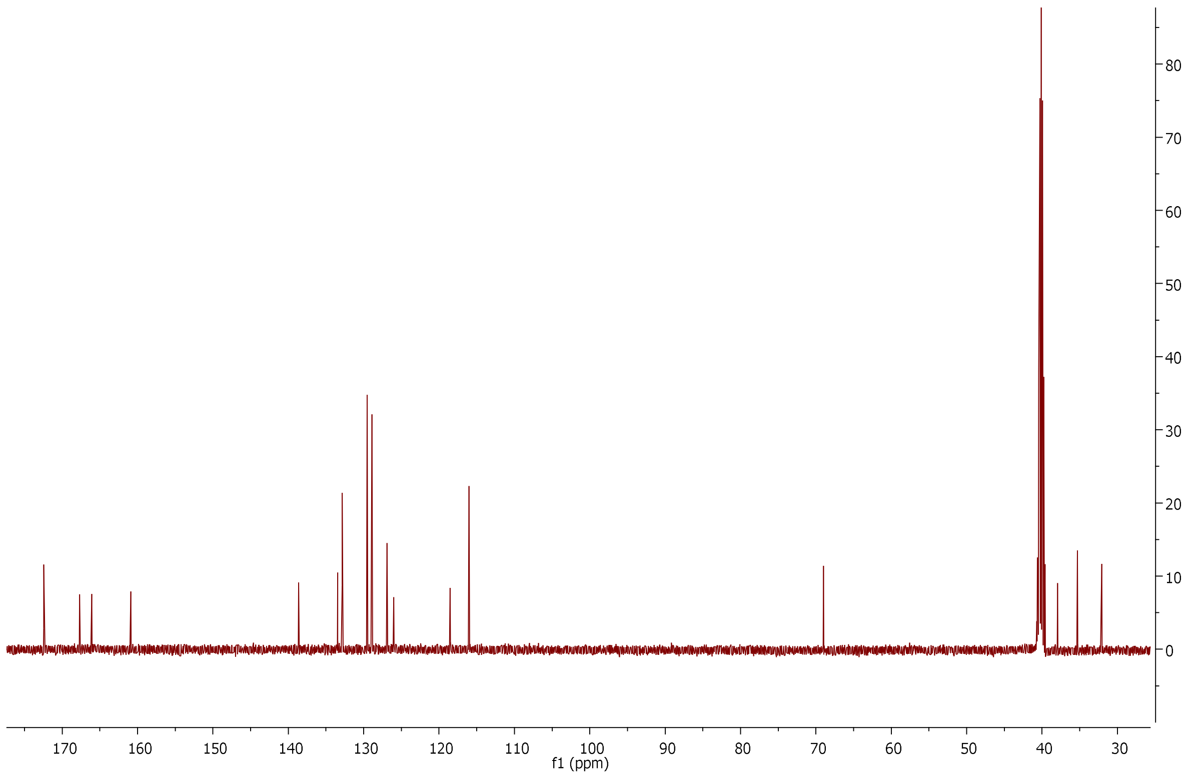

Figure S20 - ^{13}C-NMR spectrum of compound **3f** (125.73 MHz, DMSO-d_6)

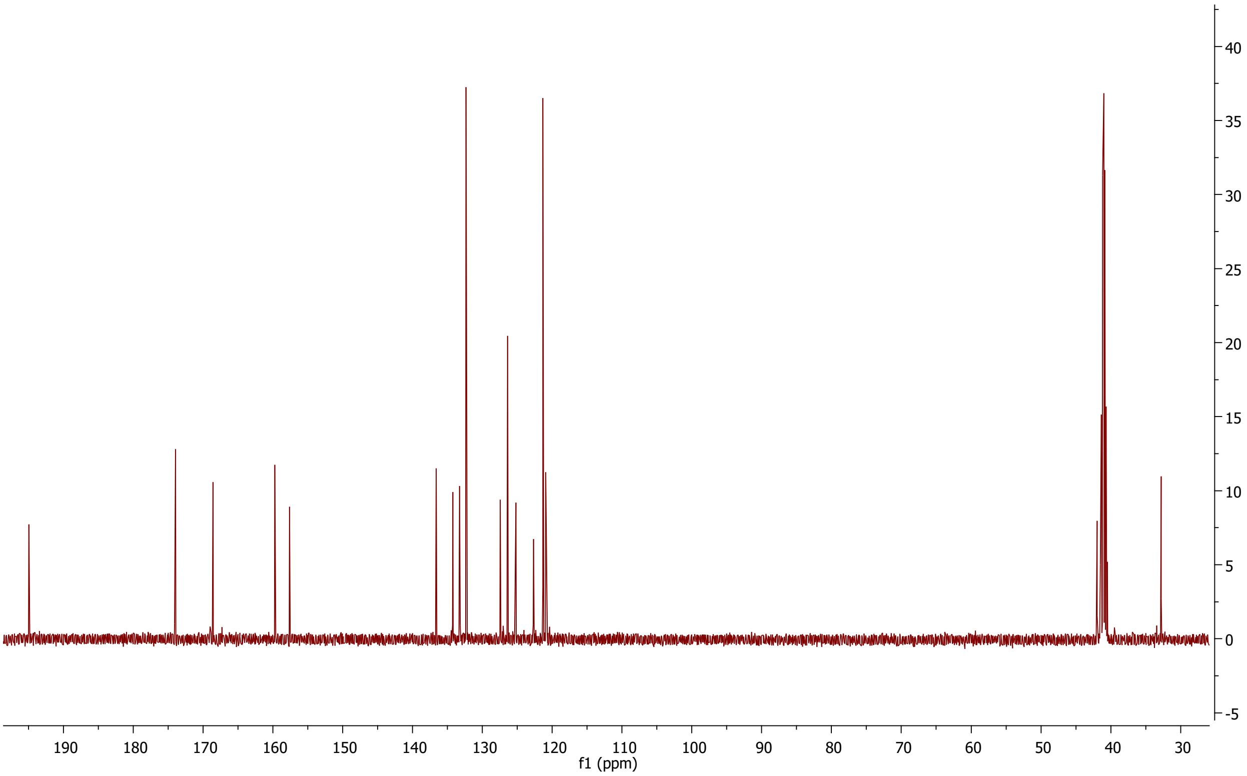

Figure S21 - ^{13}C-NMR spectrum of compound **4a** (125.73 MHz, DMSO-d_6)

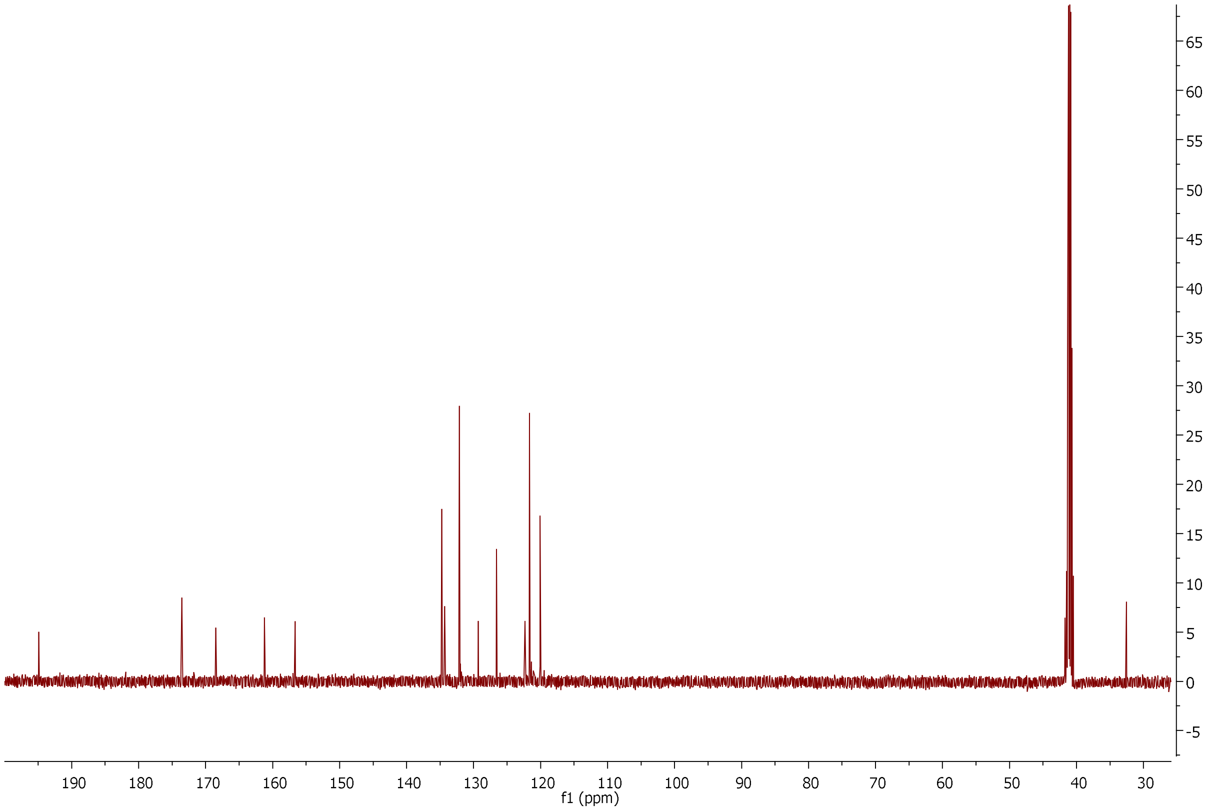

Figure S22 - ^{13}C-NMR spectrum of compound **4b** (125.73 MHz, DMSO-d_6)

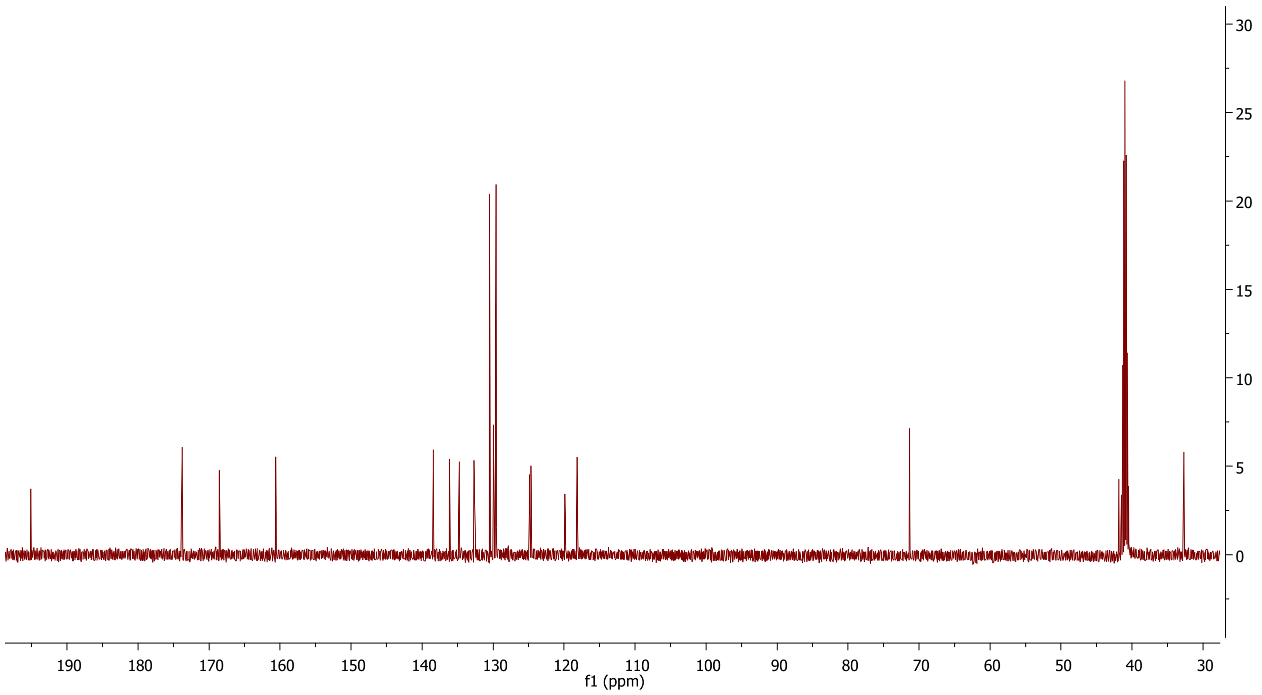

Figure S23 - ^{13}C-NMR spectrum of compound **4c** (125.73 MHz, DMSO-d_6)

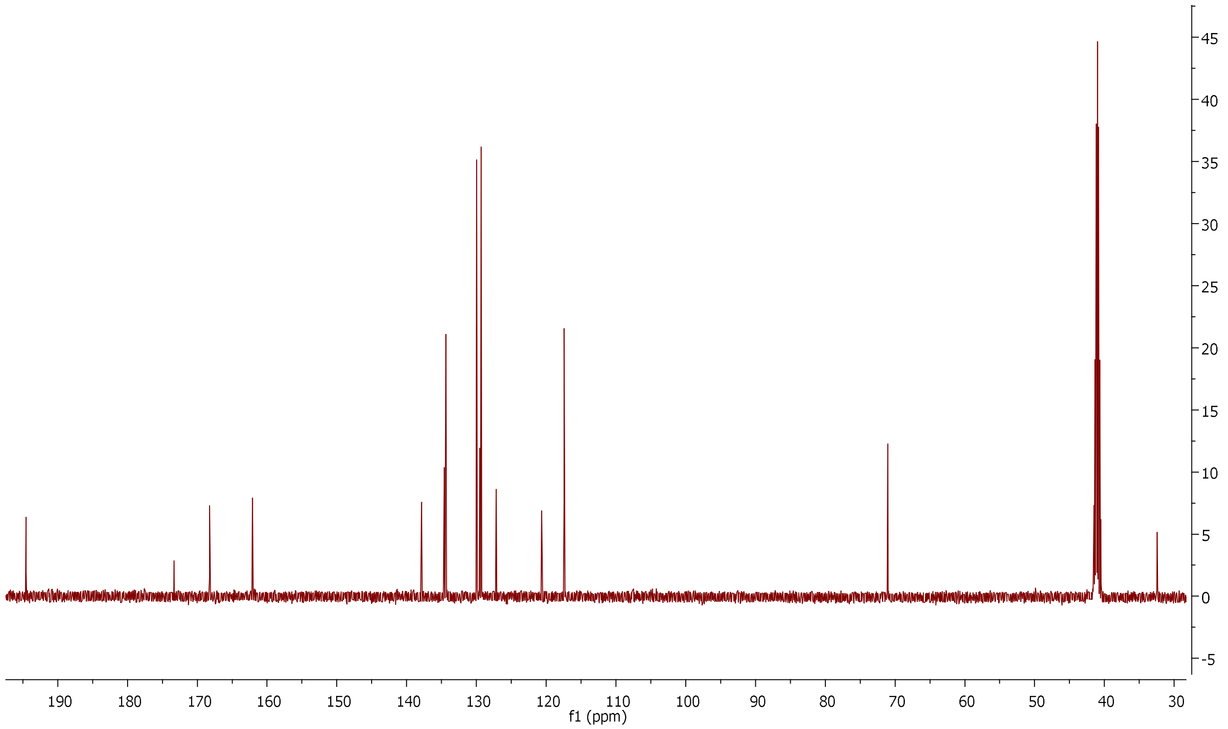

Figure S24 - ^{13}C-NMR spectrum of compound **4d** (125.73 MHz, DMSO-d_6)

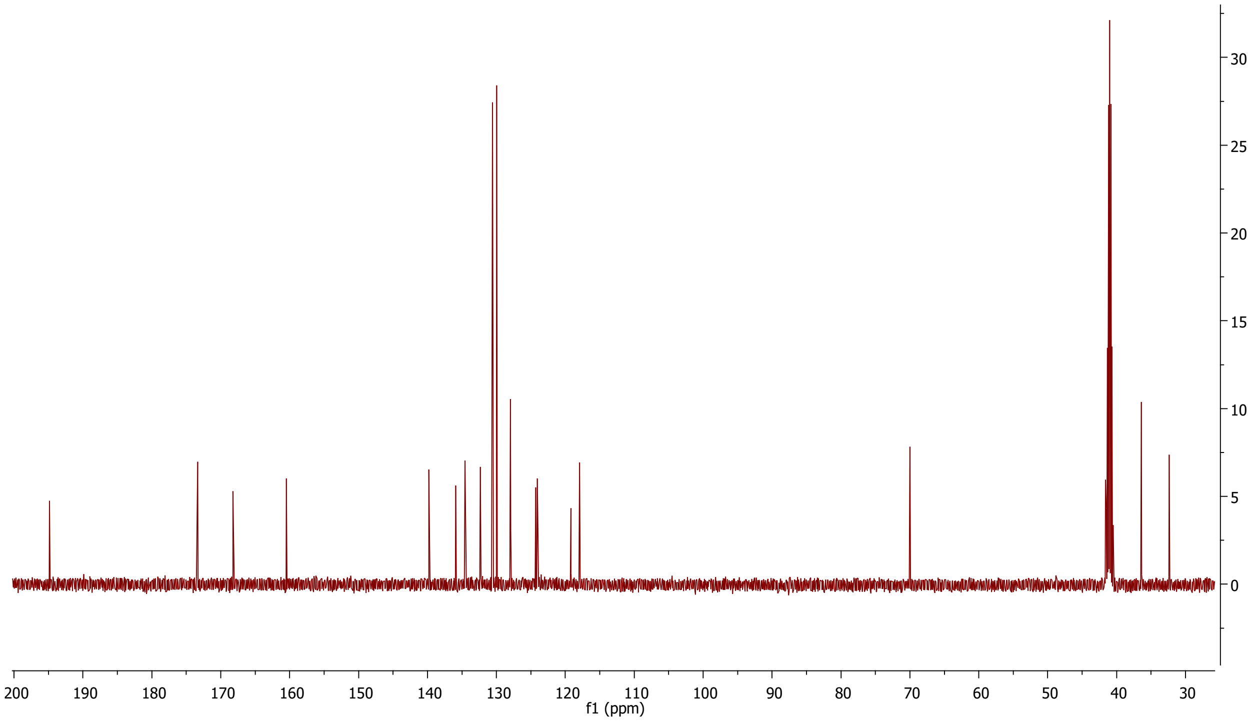

Figure S25 - ^{13}C-NMR spectrum of compound **4e** (125.73 MHz, DMSO-d_6)

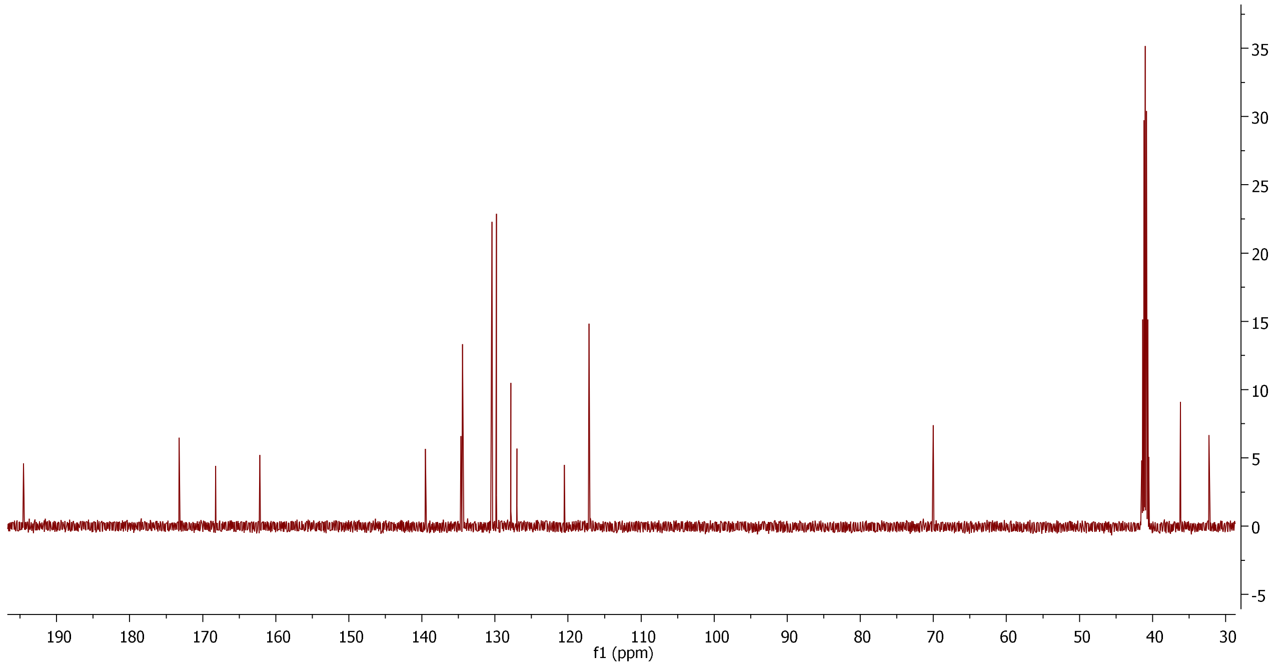

Figure S26 - ^{13}C-NMR spectrum of compound **4f** (125.73 MHz, DMSO-d_6)

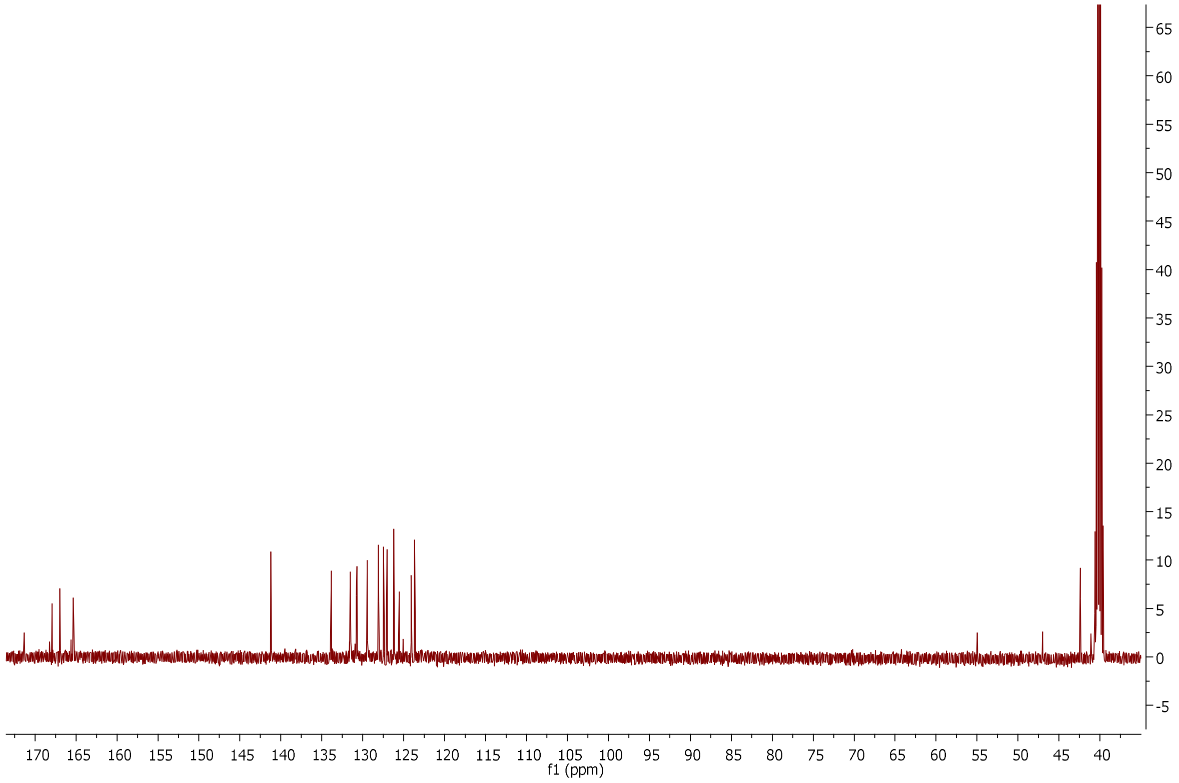

Figure S27 - ^{13}C-NMR spectrum of compound **5d** (125.73 MHz, DMSO-d_6)

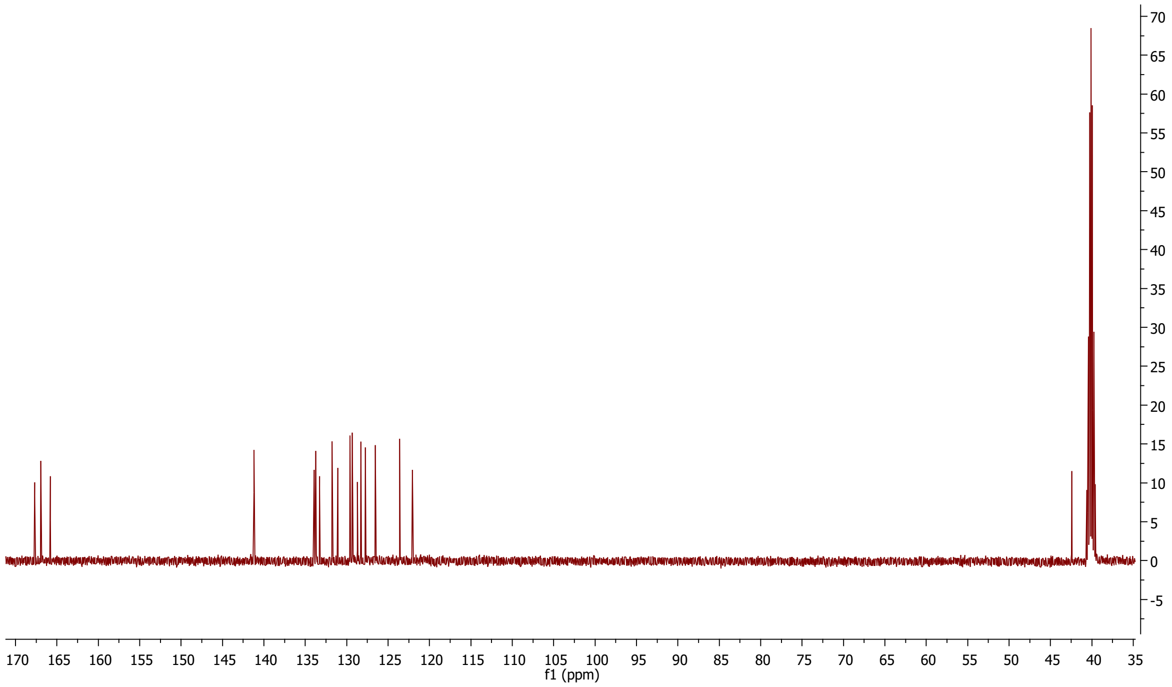

Figure S28 - ^{13}C-NMR spectrum of compound **5e** (125.73 MHz, DMSO-d_6)

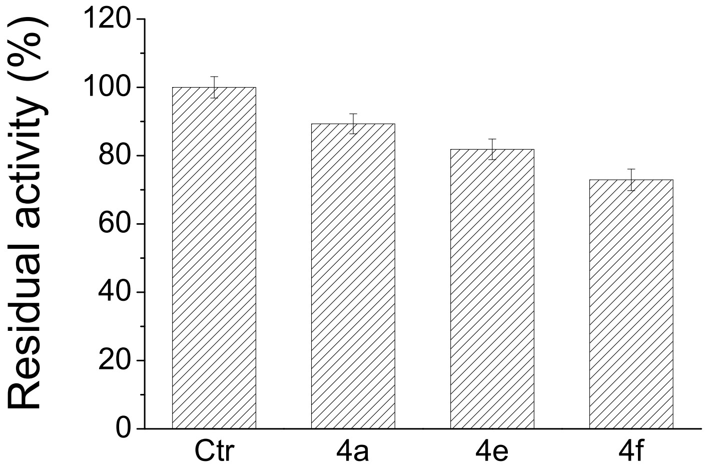

Figure S29. **Reversibility assay.** Aliquot of PTP1B was incubated in the presence of saturating concentrations of compounds **4a**, **4e** and **4f** for 1 hours at 37°C. After this time, residual activity of enzyme was determined diluting aliquot of samples in the assay buffer. All values were normalized respect to that of control experiment. Data reported in the figure represent the mean value ± S.E.M. (n = 3).

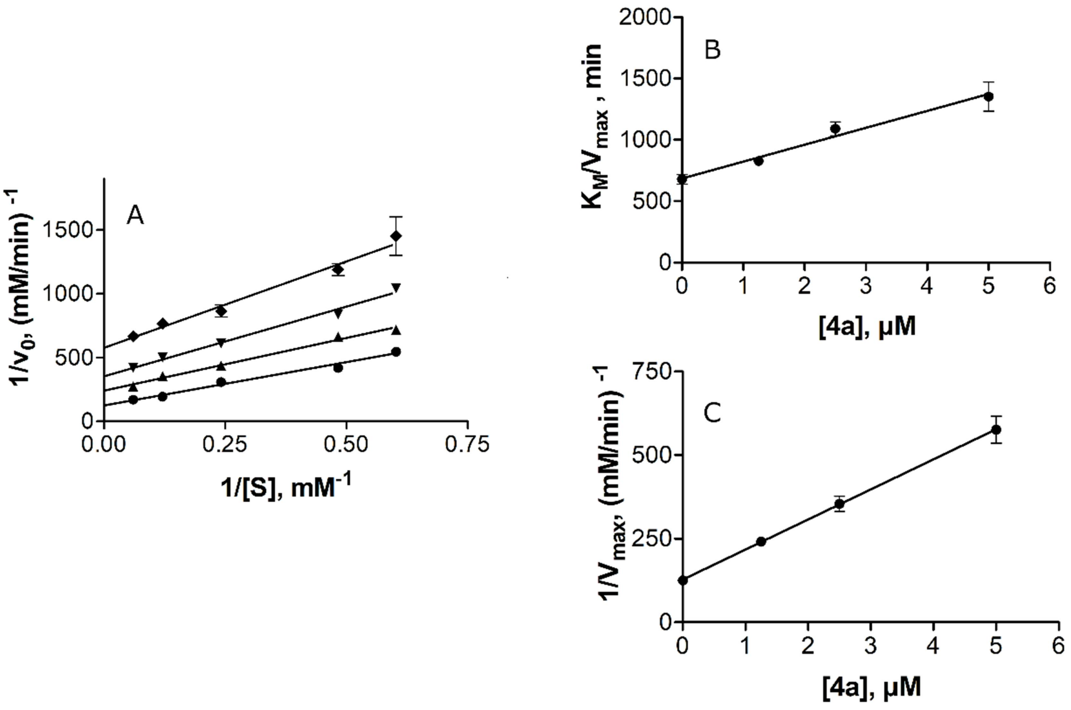

Figure S30. Kinetic characterization of compound 4a as AR inhibitor. *Panel A* refers to Linewewer-Burk plot obtained when the activity of the purified AR (8 mU) was measured at the indicated concentrations of L-idose as substrates, in the absence (●) or in the presence of the following concentrations of compound **4a**: (▲) 1.25 µM, (▼) 2.5 µM, (♦) 5 µM, *Panel* B, and *Panel C* refer to the secondary plots of the ordinate intercept ($^{app}K_M/^{app}V_{max}$) and of the slopes ($1/^{app}V_{max}$) of the relative primary plot, as a function of the inhibitor concentration. Bars (when not visible are within the symbol size) represent the standard deviations of the mean from at least three independent measurements.

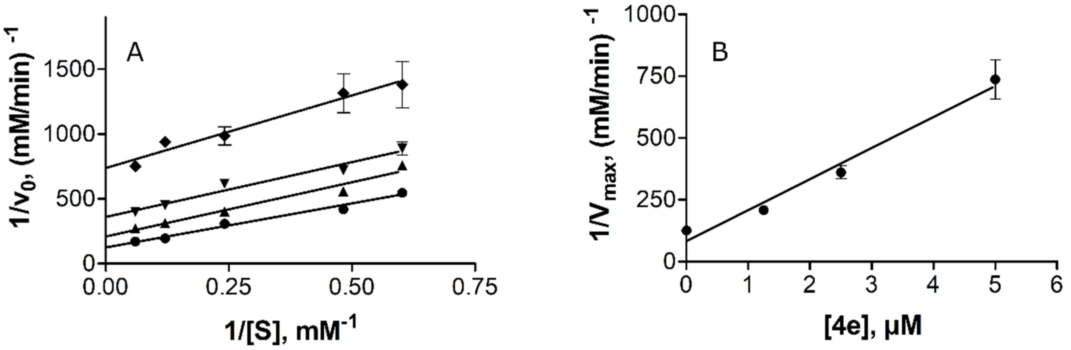

Figure S31. Kinetic characterization of compound 4e as AR inhibitor. *Panel A* refers to Linewewer-Burk plot obtained when the activity of the purified AR (8 mU) was measured at the indicated concentrations of L-idose as substrates, in the absence (●) or in the presence of the following concentrations of compound **4e**: (▲) 1.25 µM, (▼) 2.5 µM, (♦) 5 µM. *Panel* B, and *Panel C* refer to the secondary plots of the ordinate intercept ($^{app}K_M/^{app}V_{max}$) and of the slopes ($1/^{app}V_{max}$) of the relative primary plot, as a function of the inhibitor concentration. Bars (when not visible are within the symbol size) represent the standard deviations of the mean from at least three independent measurements.

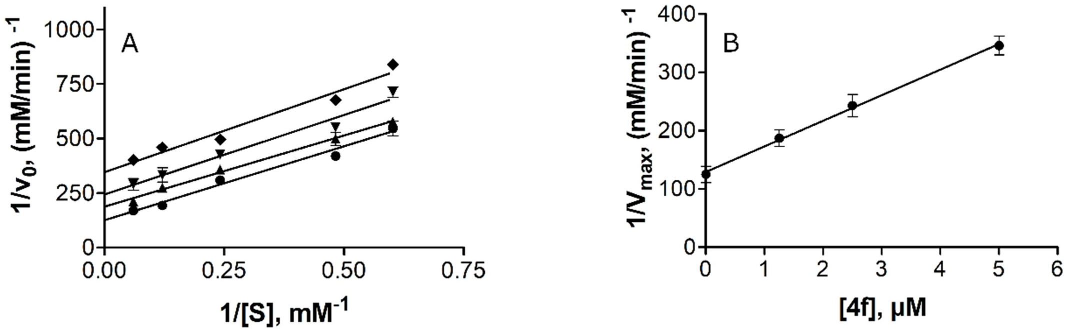

Figure S32. Kinetic characterization of compound 4f as AR inhibitor. *Panel A* refers to Linewewer-Burk plot obtained when the activity of the purified AR (8 mU) was measured at the indicated concentrations of L-idose as substrates, in the absence (●) or in the presence of the following concentrations of compound **4f**: (▲) 1.25 µM, (▼) 2.5 µM, (♦) 5 µM, *Panel* B, and *Panel C* refer to the secondary plots of the ordinate intercept ($^{app}K_M/^{app}V_{max}$) and of the slopes ($1/^{app}V_{max}$) of the relative primary plot, as a function of the inhibitor concentration. Bars (when not visible are within the symbol size) represent the standard deviations of the mean from at least three independent measurements.

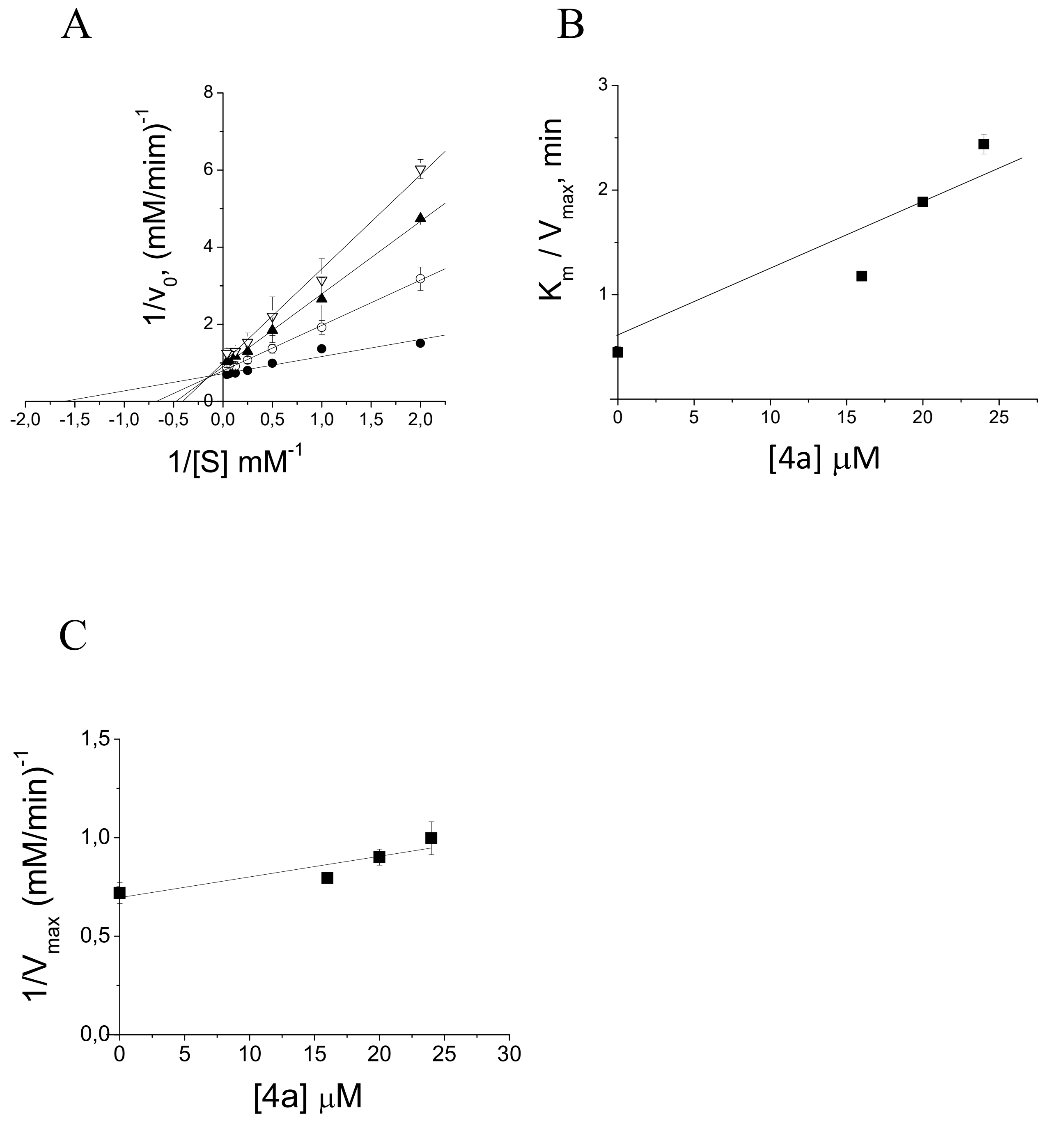

Figure S33. **Kinetic characterization of compound 4a as PTP1B inhibitor.** (A), Lineweaver–Burk plot obtained when the activity of PTP1B was measured at the indicated concentrations of pNPP in the absence (■) or in the presence of compound **4a**. The concentrations of compound **4a** used were : ○, 16 μM; ▲, 20 μM; ▽, 24 μM. Each test was carried out in triplicate. Data showed in the graph represent the mean values ± S.E.M (n = 3). Secondary plots of the slopes ($^{app}K_m$, $/^{app}V_{max}$) (B), and ($1/^{app}V_{max}$) (C), relative primary plot, as a function of the inhibitor concentration

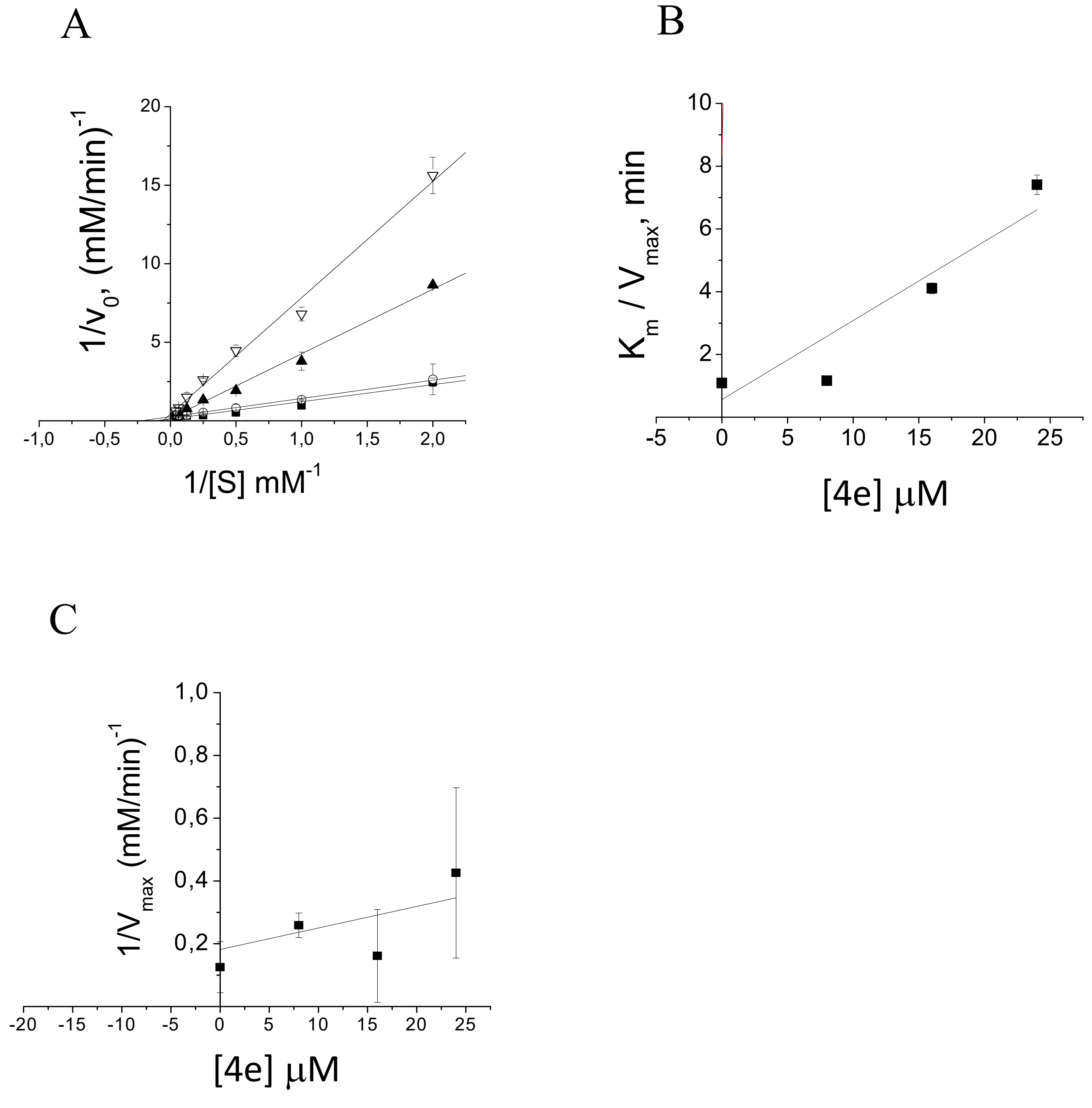

Figure S34. **Kinetic characterization of compound 4e as PTP1B inhibitor.** (A), Lineweaver–Burk plot obtained when the activity of PTP1B was measured at the indicated concentrations of pNPP in the absence (■) or in the presence of compound **4e**. The concentrations of compound **4e** used were : ○, 8 μM; ▲, 16 μM; ▽, 24 μM. Each test was carried out in triplicate. Data showed in the graph represent the mean values ± S.E.M (n = 3). Secondary plots of the slopes ($^{app}K_m$, /$^{app}V_{max}$) (B), and (1/$^{app}V_{max}$) (C), relative primary plot, as a function of the inhibitor concentration.

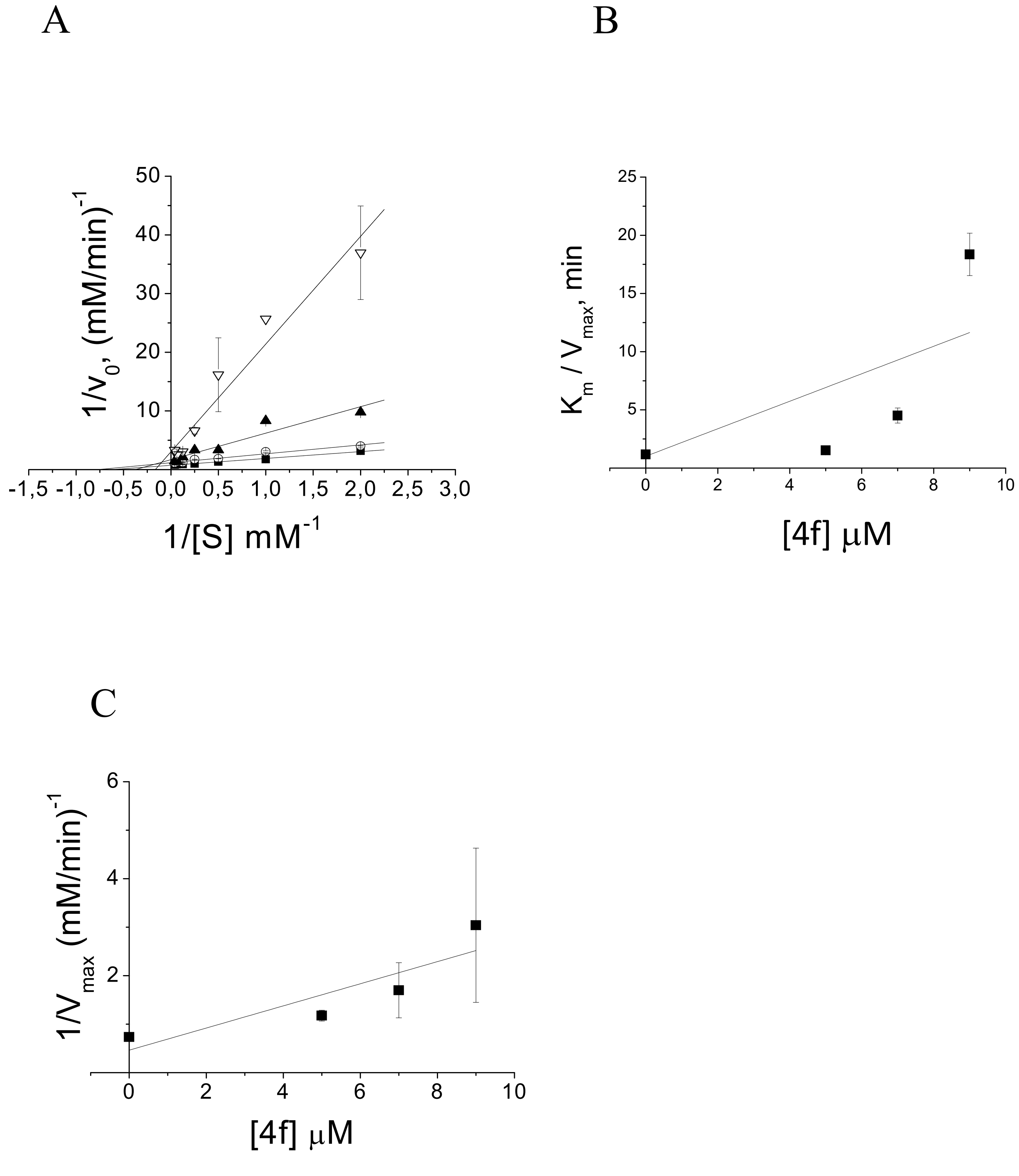

Figure S35. **Kinetic characterization of compound 4f as PTP1B inhibitor.** (A), Lineweaver–Burk plot obtained when the activity of PTP1B was measured at the indicated concentrations of pNPP in the absence (■) or in the presence of compound **4f**. The concentrations of compound **4f** used were : ○, 5 μM; ▲, 7 μM; ▽, 9 μM. Each test was carried out in triplicate. Data showed in the graph represent the mean values ± S.E.M (n = 3). Secondary plots of the slopes ($^{app}K_m$, $/^{app}V_{max}$) (B), and ($1/^{app}V_{max}$) (C), relative primary plot, as a function of the inhibitor concentration.

Table S1 – Calculated parameters of compounds **3a-f, 4a-f, 5a-e** [a]

Compd	MW	logP	n. H donor groups	n. H acceptor groups	n. rotable bonds	TPSA (A^2)
**3a**	369.39	2.83	1	5	6	109.21
**3b**	369.39	2.82	1	5	6	109.21
**3c**	383.42	2.84	1	5	7	109.21
**3d**	383.42	2.84	1	5	7	109.21
**3e**	397.44	3.06	1	5	8	109.21
**3f**	397.44	3.13	1	5	8	109.21
**4a**	385.46	3.46	1	4	6	124.23
**4b**	385.46	3.42	1	4	6	124.23
**4c**	399.48	3.36	1	4	7	124.23
**4d**	399.48	3.42	1	4	7	124.23
**4e**	413.51	3.61	1	4	8	124.23
**4f**	413.51	3.74	1	4	8	124.23
**5a**	381.40	3.05	1	5	6	109.21
**5b**	381.40	3.08	1	5	6	109.21
**5c**	365.40	3.13	1	4	5	99.98
**5d**	339.37	2.72	1	4	4	99.98
**5e**	339.37	2.71	1	4	4	99.98

[a] http://www.swissadme.ch/index.php.
